# New GABA amides activating GABA_A_-receptors

**DOI:** 10.3762/bjoc.9.42

**Published:** 2013-02-20

**Authors:** Peter Raster, Andreas Späth, Svetlana Bultakova, Pau Gorostiza, Burkhard König, Piotr Bregestovski

**Affiliations:** 1 Institute of Organic Chemistry, University of Regensburg, D-93040 Regensburg, Germany; 2 Inserm-U1106, Brain Dynamics Institute, Mediterranean University, 13005 Marseille, France; 3 Institute for Bioengineering of Catalonia (IBEC) and Institució Catalana de Recerca i Estudis Avançats (ICREA), 08028 Barcelona, Spain

**Keywords:** CHO-cells, GABA, GABA-amides, GABA-superagonist, patch-clamp recording

## Abstract

We have prepared a series of new and some literature-reported GABA-amides and determined their effect on the activation of GABA_A_-receptors expressed in CHO cells. Special attention was paid to the purification of the target compounds to remove even traces of GABA contaminations, which may arise from deprotection steps in the synthesis. GABA-amides were previously reported to be partial, full or superagonists. In our hands these compounds were not able to activate GABA_A_-receptor channels in whole-cell patch-clamp recordings. New GABA-amides, however, gave moderate activation responses with a clear structure–activity relationship suggesting some of these compounds as promising molecular tools for the functional analysis of GABA_A_-receptors.

## Introduction

**Figure 1:**
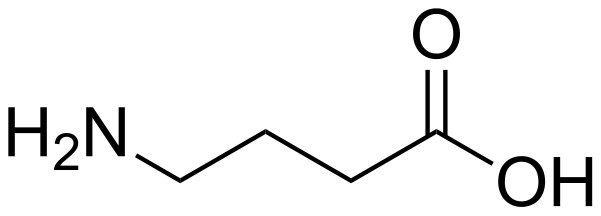
The neurotransmitter GABA.

γ-Aminobutyric acid (GABA) is the major inhibitory amino acid transmitter of the central nervous system (CNS) of vertebrates (Figure 1).

It plays an important role in a variety of physiological functions, including motion control, vision, relaxation, sleep and many other brain functions [1–3]. In vertebrates GABA activates specific receptors of several classes: GABA_A_, GABA_B_ and GABA_C_ [4]. GABA_A_-receptors are transmembrane heterooligomeric proteins, forming chloride (Cl^−^) selective channels, and are composed of five subunits: two α, two β and one γ subunit [5]. Being highly expressed in the peripheral and central nervous system, GABA_A_-receptors represent a key therapeutic target for benzodiazepines, barbiturates, neurosteroids and general anesthetics [6–8]. Therefore, in spite of a large variety of existing agonists, antagonists and modulators of GABA-receptors, there is a high interest in the development of new drugs that can interact with these targets. Many compounds of different substance classes are known to modulate the activity of GABA_A_-receptors [9–12]. One substance group that has been less explored is GABA-amides. Compounds **4a**, **4b**, **4c, 7a** (as triflate salt) and **7c** were previously reported to potently activate the GABA_A_-channels, being "partial, full or superagonists" [13]. However, in their work the authors studied the activity of GABA-amides on GABA_A_-receptors using chloride-flux assays on synaptoneurosomes. Such preparations can contain many damaged cells leading to a highly variable intracellular Cl^−^ distribution in different cells. In our study we used the patch-clamp technique, which is much more reliable and informative in comparison to the approach based on chloride-flux assays. Like Carlier et al. [13], we noticed that in the course of the GABA-amide synthesis, GABA impurities are generated in the deprotection step. However, in contrast to Carlier et al. [13], who used a modification of Sallers’s procedure [14] (detection limit <0.1 wt %) to check the purity of the compounds, we developed an improved purification procedure and used a more sensitive HPLC–MS analysis to ensure that the GABA-amide products do not contain any detectable amount of GABA (detection limit <0.002 wt %). Our observations may help to give a more complete picture of the ability of GABA-amides to activate GABA_A_ receptors.

## Results and Discussion

**Scheme 1:**
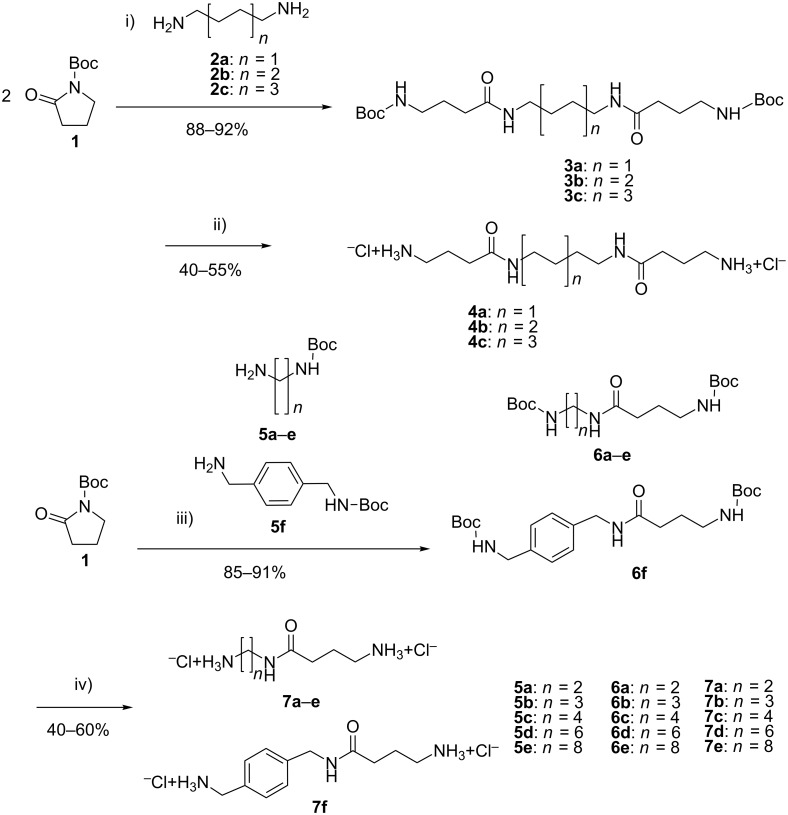
Synthesis of GABA-amide hydrochlorides **4a**–**c** and **6a**–**f**. (i) THF, reflux; (ii) MeOH, HCl (5%); (iii) THF, reflux; (iv) MeOH, HCl (5%).

Boc-Pyrrolidone **1** was nucleophilic-ring-opened by diamines **2a**–**c** or the mono Boc-protected diamines **5a**–**f** in THF (Scheme 1, See Supporting Information File 1 for full experimental data).

To cleave the Boc-protecting groups, the compounds were dissolved in dilute HCl (5%, EtOH/H_2_O). It was observed that the use of more concentrated HCl can cause cleavage of the amide bonds and the release of free GABA. To remove any impurities of GABA, which may lead to false test results, all substances were carefully purified: In a first purification step the GABA-amide hydrochlorides were dissolved in MeOH and precipitated by slow addition of Et_2_O. The precipitates were centrifuged and recrystallized several times from MeOH until no traces of GABA could be detected by HPLC–MS analysis. To determine the sensitivity of the analysis method three stock solutions of GABA were prepared (10^−3^, 10^−5^ and 10^−7^ mol/L) and 1 µL was injected into the HPLC-coupled mass spectrometer. On the basis of the obtained mass spectra, a GABA detection limit of 0.14 pmol (14 pg) was determined.

**Figure 2:**
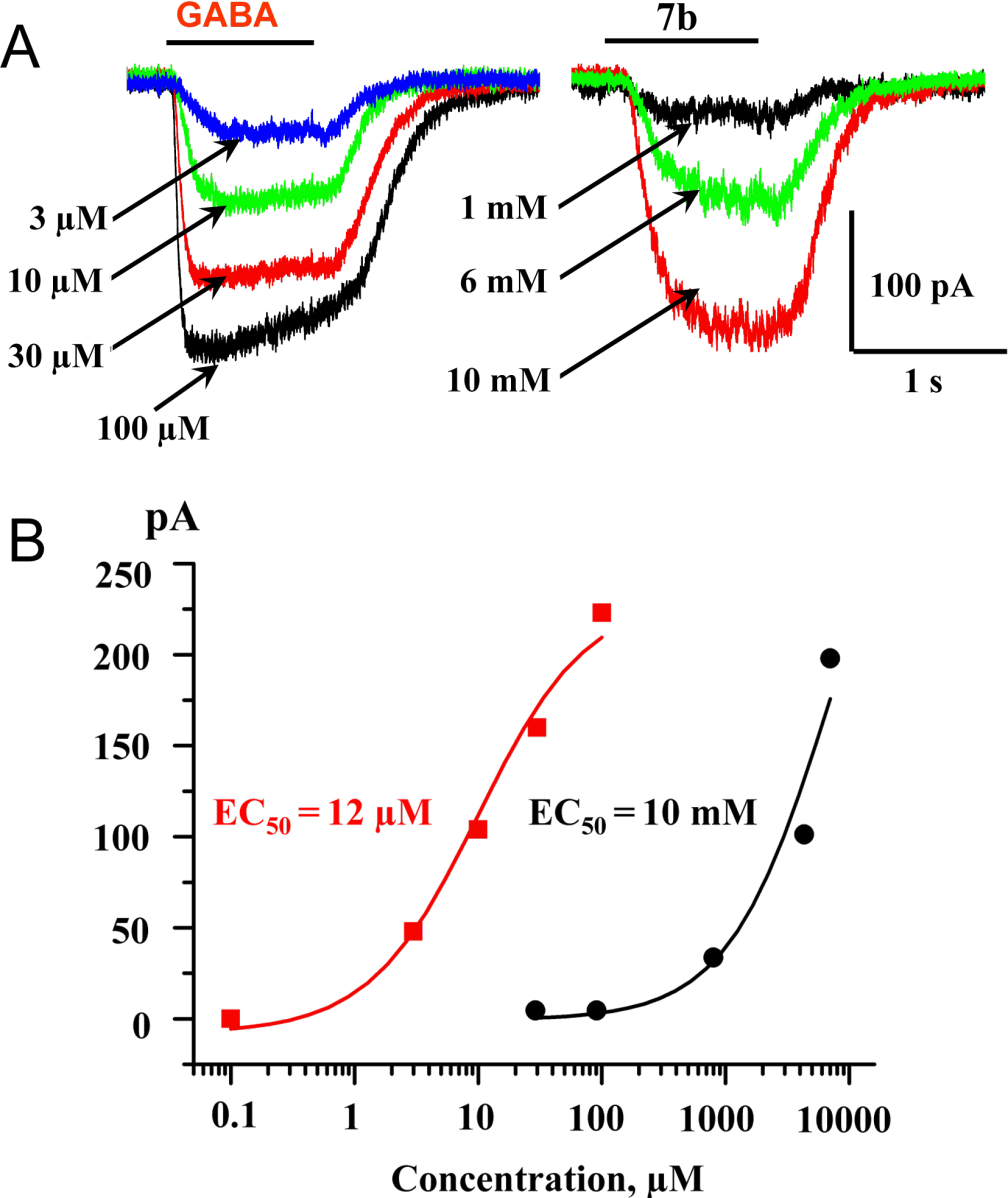
Effect of **7b** on GABA_A_-receptor activation. (A) Superimposed traces of whole-cell currents induced by rapid application of GABA (left) or compound **7b** (right) in CHO cells transfected with α_1_-GFP/β/γ_2L_ combination of GABA_A_ receptor subunits; (B) Concentration dependencies of GABA (closed squares) and **7b** (closed circles). EC_50_s were 12 µM and 10 mM for GABA and **7b**, respectively.

By examining the Cl^−^ uptake elicited by different GABA_A_-receptor agonists, Carlier et al. demonstrated that compounds with general structure **4a**–**c** were capable of stimulating Cl^−^ uptake with different efficacy [13]. Moreover, the authors described compound **4b** as "superagonist", because it induced a maximum uptake about 50% higher than that achieved by GABA. Surprisingly, the EC_50_ value for this compound was more than 30-fold higher than for GABA, i.e., at 733 µM and 14.3 µM, respectively. The investigations in this study were performed by using a standard ^36^Cl^−^-flux assay in mouse brain synaptoneurosomes. This technique does not allow a comparative analysis on one cell. Moreover, ^36^Cl^−^-flux assays were carried out by using a 15 s incubation time. As GABA-receptors usually exhibit strong desensitization at long exposures to agonists, we decided to re-examine these results using patch-clamp recordings and fast perfusion technique for the application of the tested compounds. The activity of synthesized compounds was tested by using CHO cells transiently expressing GABA_A_-receptors in the configuration (α_1_-GFP + β_2_ + γ_2Long_). To analyze the functional properties of the compounds, we performed monitoring of ionic currents using whole-cell patch-clamp techniques. First, concentration-response curves for GABA were obtained and its EC_50_ was determined. Then we applied different concentrations of the studied compounds and estimated the minimal concentration that induced currents and, if possible, their EC_50_. The EC_50_ for GABA varied in different cells from 4 µM to 15 µM with a mean of 9.5 ± 0.3 µM (*x* [number of tested cells] = 10) (Figure 2, see Supporting Information File 1 for full experimental data).

**Figure 3:**
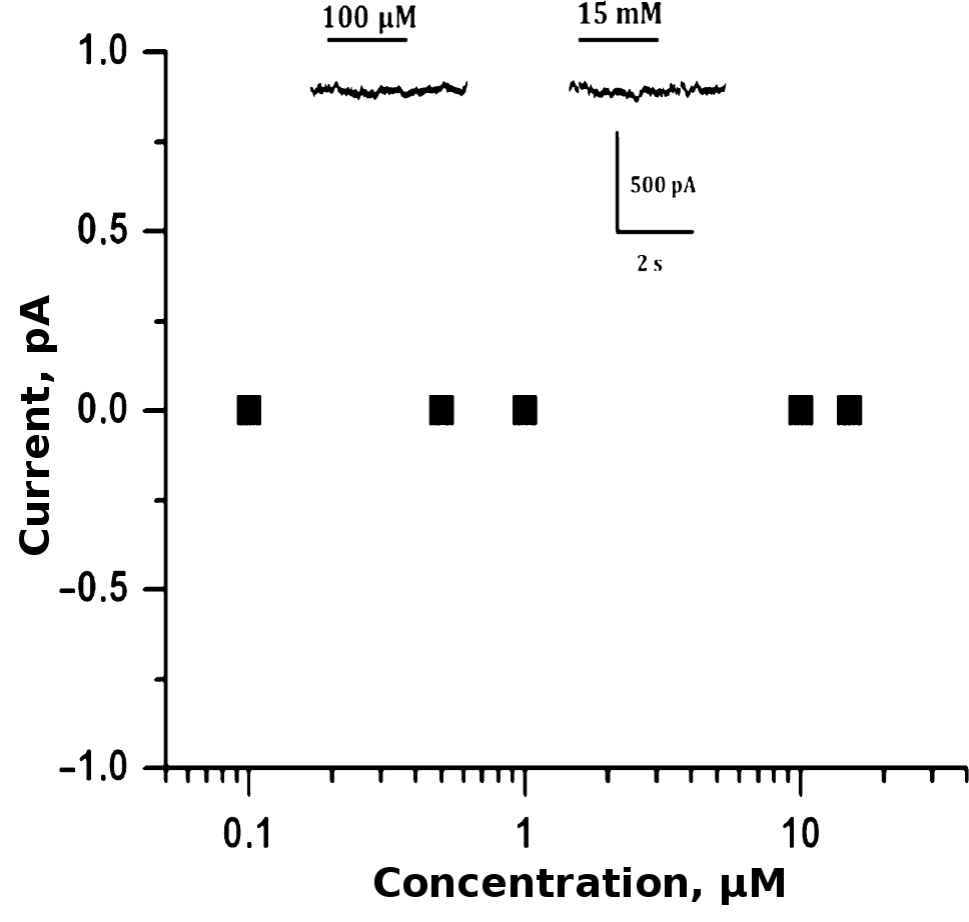
Absence of GABA_A_-receptor activation on application of **4c**. Top traces: Examples of whole-cell currents on rapid application of **4c** on CHO cells transfected with an α_1_-GFP/β_2_/γ_2L_ combination of GABA_A_-receptor subunits. Note that the compound was not able to induce currents even at a concentration of 15 mM.

Surprisingly, in contrast to previously described observations, our purified compounds **4a**–**c** were not able to activate GABA_A_-receptors in concentrations up to 10 mM. Figure 3 illustrates this for the compound **4c**. Similar results were obtained also for compounds **4a** and **4b**: on application of 10 mM, the changes in the current were 0 pA, (*x* = 4 for each compound).

**Figure 4:**
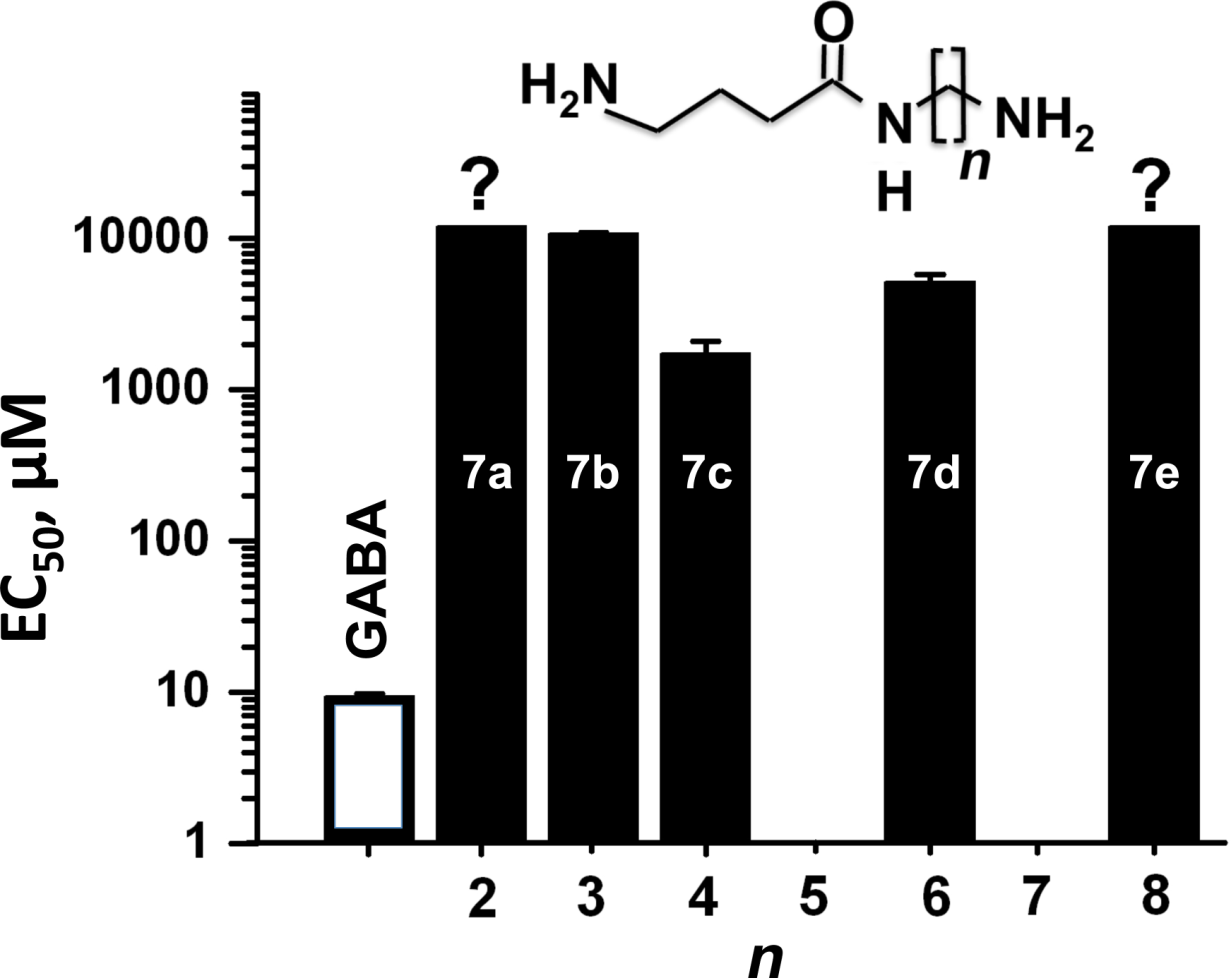
Variation of EC_50_ values with the number *n* of methylene units separating the amide and the ammonium group in compounds **7a**–**e**. Each column represents results from 3–7 cells. The mark [?] on the top of bars indicates that for the EC_50_‘s for these compounds were not determined precisely.

In contrast, all studied compounds from series **7a**–**e** were capable of inducing ionic currents with different efficacy (Figure 4).

**Figure 5:**
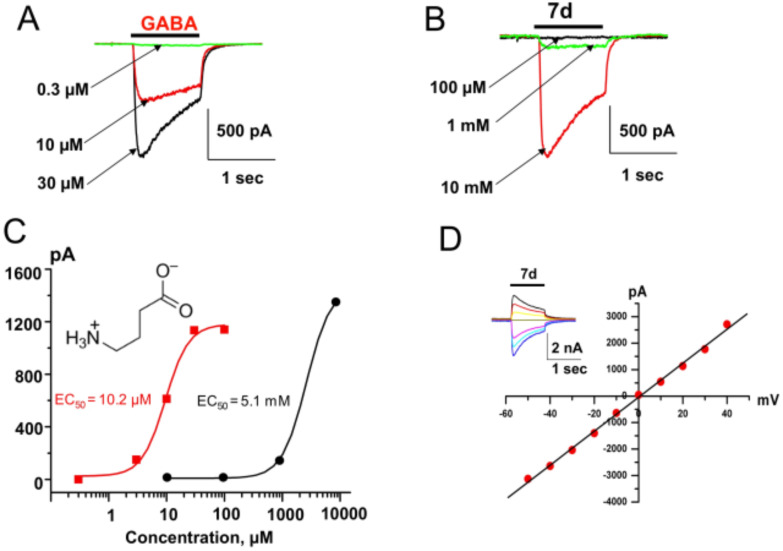
Effect of **7d** on GABA_A_-receptor activation. (A,B) Superimposed traces of whole-cell currents induced by rapid application of GABA (A) or the compound **7d** (B) in CHO cells transfected with an α_1_-GFP/β_2_/γ_2L_ combination of GABA_A_-receptor subunits; (C) Concentration dependencies obtained on application of GABA (red closed square) and **7d** (black closed circles). EC_50_ values were 10.2 µM and 5.1 mM for GABA and **7d**, respectively; (D) Current–voltage relations for responses induced by compound **7d**. Insert: examples of traces at different membrane potentials. Scales: 2 nA and 1 s.

Thus, compound **7a** (*n* = 2) activated currents at 2.6 mM with an amplitude of 10–30 pA (*x* = 4). The compound **7b** (*n* = 3) activated GABA-receptors more strongly and at concentrations of 10 mM induced currents comparable to those for GABA 30–100 µM (Figure 2). The EC_50_ for compound **7b** is 1080 ± 140 µM (*x* = 4). The efficacy of the compounds is significantly weaker than for GABA. The number of -CH_2_-units between the amide nitrogen atom and the ammonium moiety of compounds **7** affects the efficacy significantly, reaching a maximum with compound **7c** (*n* = 4). The EC_50_ for compound **7c** is 1750 ± 330 µM (*x* = 7). Compound **7d** (*n* = 6) also effectively activated GABA_A_-receptors with an activation threshold of about 100 µM. At concentrations of 10 mM it caused currents similar of those induced by saturated GABA concentrations (30–100 µM) with an EC_50_ of about 5 mM (Figure 5A–C). The EC_50_ for compound **7d** is 5250 ± 560 µM (*x* = 6). Kinetics of desensitization and the current–voltage dependencies (Figure 5D) of the studied compounds are similar to those for GABA. Compound **7e** (*n* = 8) weakly activated currents at 5 mM with an amplitude of 10–20 pA (*x* = 3).

## Conclusion

Our data suggest that compounds **4a**–**c** are not capable of activating GABA_A_-receptors. Compounds **7a**–**e** are able to stimulate these receptors and show a distinct structure–activity correlation. The compounds may become useful as molecular tools for the functional analysis of GABA_A_-receptors.

## Supporting Information

Exact synthetic procedures, copies of ^1^H NMR and HPLC–MS spectra of GABA-amide hydrochlorides **4a**–**c** and **7a**–**f**. Cell culture, transient transfection methods and electrophysical recordings.

File 1 Experimental part.
