# Supplementary material for: New GABA amides activating GABAA-receptors
Source: Beilstein J Org Chem. 2013 Feb 20;9:406–10. doi: 10.3762/bjoc.9.42 (PMC3596083; doi:10.3762/bjoc.9.42)
Supplement: File 1 — Experimental part. [file Beilstein_J_Org_Chem-09-406-s001.pdf]

Supporting Information

for

New GABA amides activating $GABA_A$ receptors

Peter Raster[1], Andreas Späth[1], Svetlana Bultakova[2], Pau Gorostiza[3], Burkhard König[*1] and Piotr Bregestovski[*2]

Address: [1]Institute of Organic Chemistry, University of Regensburg, D-93040 Regensburg, Germany, [2]Inserm-U1106, Brain Dynamics Institute, Mediterranean University, 13005 Marseille, France and [3]Institute for Bioengineering of Catalonia (IBEC) and Institució Catalana de Recerca i Estudis Avançats (ICREA), 08028 Barcelona, Spain.

Email: Burkhard König - burkhard.koenig@chemie.uni-regensburg.de, Piotr Bregestovski - piotr.bregestovski@univmed.fr

* Corresponding author

Experimental part

		page
I.	Chemistry	S2
I.I	Methods and synthesis	S2
I.II	NMR and mass spectra	S10
II.	Biology	S22
II.I	Methods	S22
III.	References	S23

I. Chemistry

I.I Methods and synthesis

The following compounds were prepared according to literature methods: *tert*-butyl 2-oxopyrrolidine-1-carboxylate [1], *tert*-butyl (2-aminoethyl)carbamate [2], *tert*-butyl (3-aminopropyl)carbamate [3], *tert*-butyl (4-aminobutyl)carbamate [4], *tert*-butyl (6-aminohexyl)carbamate [3], *tert*-butyl (8-aminooctyl)carbamate [3], *tert*-butyl 4-(aminomethyl)benzylcarbamate [5]. All other reagents were obtained from commercial sources. Unless otherwise noted, solvents (analytical grade) were purchased from commercial suppliers and used without further purification. Melting points were obtained using a Lambda Photometrics Optimelt MPA 100 apparatus (Lambda Photometrics Harpenden, UK) and are not corrected. IR spectra were obtained using a Varian 3000 spectrometer. ^{1}H NMR spectra were recorded at 300 MHz on a Bruker Avance 300 spectrometer. ^{13}C NMR spectra were recorded at 75 MHz on a Bruker Avance 300 spectrometer. The NMR spectra were recorded in $CDCl_3$, DMSO or MeOD as solvent and chemical shifts are reported in ppm. Mass spectra were obtained using Finnigan SSQ 710A (EI), Finnigan MAT 95 (CI) or Finnigan MAT TSQ 7000 (Thermo FINNIGA, USA) (ES/LS-MS)] instrumentation. Thin-layer chromatography (TLC) was performed on alumina plates coated with silica gel (Merck silica gel 60 F_{245}, thickness 0.2 mm). Column chromatography was accomplished with Merck Geduran SI 60 silica gel as the stationary phase. Petroleum ether (PE) refers to the fraction boiling in the 30–60 °C range.

General procedure (GP 1) for the synthesis of Boc-protected GABA-amides (**3a–c**): Boc-pyrrolidone **1** (5 mmol, 0.86 g) was dissolved in abs. THF (5 mL) and the diamines **2a–c** (2.5 mmol) were added. The solution was stirred under reflux and nitrogen atmosphere until the diamines were completely converted as monitored by TLC. The solvent was evaporated,

the crude product was purified by flash chromatography (EA), and white solids were obtained.

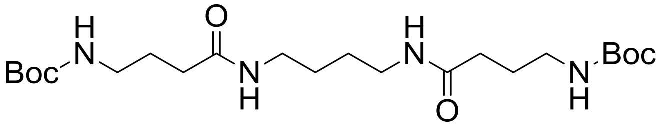

Di-*tert*-butyl ((butane-1,4-diylbis(azanediyl))bis(4-oxobutane-4,1-diyl))dicarbamate (**3a**) [6]: The compound was prepared according to GP 1 yielding 1.05 g (92%) of the target product.

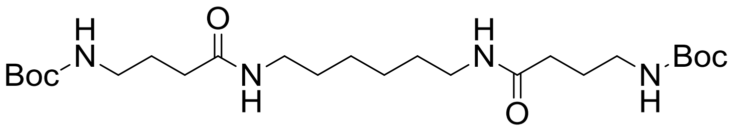

Di-*tert*-butyl ((hexane-1,6-diylbis(azanediyl))bis(4-oxobutane-4,1-diyl))dicarbamate (**3b**) [6]: The compound was prepared according to GP 1 yielding 90% (1.09 g) of the target product.

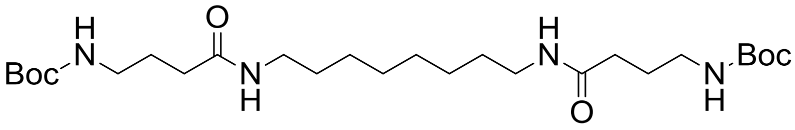

Di-*tert*-butyl ((octane-1,8-diylbis(azanediyl))bis(4-oxobutane-4,1-diyl))dicarbamate (**3c**) [6]: The compound was prepared according to GP 1 yielding 88% (1.10 g) of the target product.

General procedure (GP 2) for the Boc deprotection (**4a–c**): The Boc protected diamines **3a–c** (1 mmol) were dissolved in EtOH (10 mL), a HCl solution (5%, 20 mL) was added over a period of 2–3 h, and the reaction progress was monitored by TLC. A second portion of aqueous HCl (0.5 M, 100 mL) was added if necessary, and the solution was stirred until the deprotection was completed. EtOH was evaporated and the remaining H_2O was removed by lyophilisation. The product was recrystallised from MeOH twice. Drying in vacuo afforded the deprotected amine hydrochlorides as colorless, white solids.

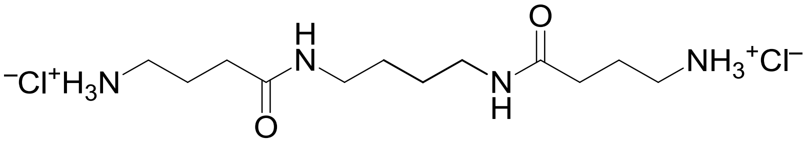

N,*N*´-(Butane-1,4-diyl)bis(4-aminobutanamide) dihydrochloride (**4a**) [6]: The compound was prepared according to GP 2 yielding 48% (168 mg) of the target compound.

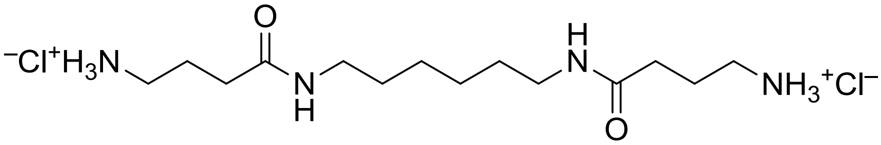

N,*N*´-(Hexane-1,6-diyl)bis(4-aminobutanamide) dihydrochloride (**4b**) [6]: The compound was prepared according to GP 2 yielding 55% (179 mg) of target compound **4b**.

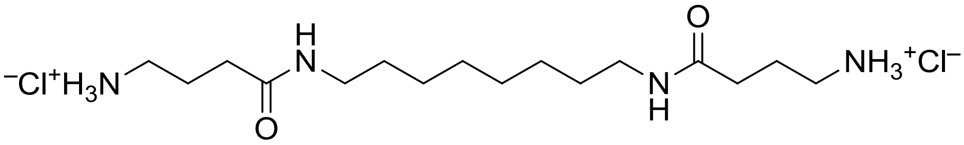

N,*N*´-(Octane-1,8-diyl)bis(4-aminobutanamide) dihydrochloride (**4c**) [6]: The compound was prepared according to GP 2 yielding 40% (154 mg) of the target compound.

General procedure (GP 3) for the preparation of the Boc-protected GABA-amides (**6a–f**): N-Boc-pyrrolidone **1** (1.85 g, 10 mmol) and the mono Boc-protected amines (**5a–f**) (15 mmol) were dissolved in THF (10 mL), and the solution was heated under reflux for 2 d. The solvent was evaporated and the residue was dissolved in dichloromethane (30 mL). The organic layer was washed with H_2O (20 mL) and brine (20 mL), dried over $MgSO_4$ and the solvent was evaporated. The raw material was purified by column chromatography (EA:PE = 1:1 → 3:1) to give colorless solids.

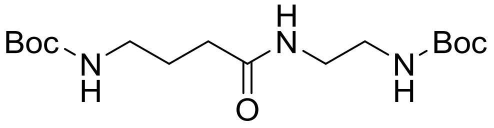

4-(*tert*-Butyloxycarbonylamino)-*N*-(2-(*tert*-butyloxycarbonylamino)ethyl)butanamide (**6a**) [6]: The compound was prepared according to GP 3 yielding 91% (3.10 g) of **6a**.

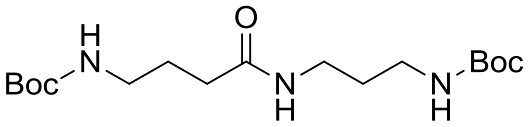

4-(*tert*-Butyloxycarbonylamino)-*N*-(3-(*tert*-butyloxycarbonylamino)propyl)butanamide (**6b**): The compound was prepared according to GP 3 yielding 88% (3.15 g) of **6b** as a white solid, mp 80–82 °C. ^{1}H NMR (300 MHz, $CDCl_3$) δ 1.27–1.30 (m, 4H), 1.40–1.57 (m, 14H), 1.64–1.87 (m, 4H), 2.18 (t, 2H, *J* = 6.8 Hz), 3.04–3.26 (m, 6H), 4.47 (s, 1H), 4.80 (s, 1H), 6.20 (s, 1H) ppm; ^{13}C NMR (75 MHz, $CDCl_3$) δ 26.8, 27.5, 27.6, 28.8, 30.4, 34.4, 40.8, 41.4, 79.4, 79.6, 155.8, 155.9, 172.5 ppm; IR: ν_{max} = 3430, 3230, 3080, 3050, 2940, 2970, 2870, 2803, 2744, 2635, 2383, 2340, 2032, 1779, 1682, 1611, 1539, 1482, 1386, 1349, 1310, 1284, 1260, 1213, 1152, 1068, 1019, 976; HRMS (ESI): calcd. for $C_{17}H_{33}N_3O_5$ 359.2420; found 359.2425.

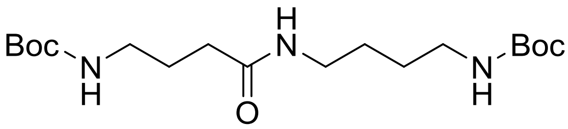

4-(*tert*-Butyloxycarbonylamino)-*N*-(4-(*tert*-butyloxycarbonylamino)butyl)butanamide (**6c**) [6]: The compound was prepared according to GP 3 yielding 86% (3.06 g) of **6c**.

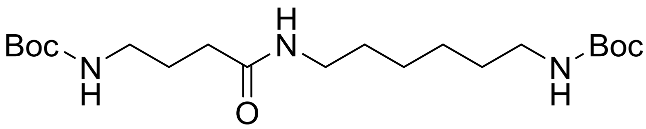

4-(*tert*-Butyloxycarbonylamino)-*N*-(6-(*tert*-butyloxycarbonylamino)hexyl)butanamide (**6d**): The compound was prepared according to GP 3 yielding 90% (3.21 g) of **6d** as a white solid, mp 82–84 °C. ^{1}H NMR (300 MHz, $CDCl_3$) δ 1.27–1.31 (m, 4H), 1.40–1.55 (m, 20H), 1.66–1.85 (m, 4H), 2.20 (t, 2H, *J* = 6.9 Hz), 3.04–3.28 (m, 6H), 4.50 (s, 1H), 4.77 (s, 1H), 6.18 (s,

1H) ppm; ^{13}C NMR (75 MHz, $CDCl_3$) δ 26.8, 27.5, 27.7, 28.8, 30.4, 30.9, 34.5, 40.4, 40.9, 41.3, 41.5, 79.5, 79.6, 155.8 155.9, 172.6 ppm; IR: v_{max} = 3437, 3231, 3085, 3053, 2944, 2972, 2874, 2803, 2744, 2629, 2585, 2383, 2335, 2032, 1779, 1682, 1611, 1539, 1482, 1386, 1349, 1310, 1284, 1263, 1213, 1152, 1068, 1019, 976, 909; HRMS (ESI): calcd. for $C_{20}H_{39}N_3O_5$ 401.2890; found 401.2895.

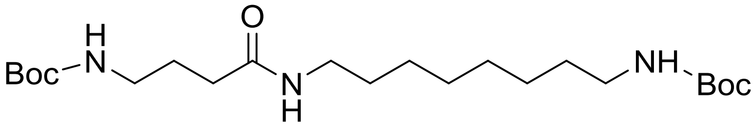

4-(*tert*-Butyloxycarbonylamino)-*N*-(8-(*tert*-butyloxycarbonylamino)octyl)butanamide (**6e**): The compound was prepared according to GP 3 yielding 85% (3.65 g) of **6e** as a white solid, mp 92–94 °C. ^1H NMR (300 MHz, $CDCl_3$) δ 1.41–1.51 (m, 26H), 1.75–1.85 (m, 4H), 2.16–2.23 (d, 2H, J = 6.9 Hz), 3.05–3.26 (m, 8H), 4.52 (s, 1H), 4.77 (s, 1H), 6.16 (s, 1H) ppm; ^{13}C NMR (75 MHz, $CDCl_3$) δ 27.5, 27.7, 28.3, 28.4, 29.3, 29.4, 26.7, 26.8, 26.4, 26.2, 39.2, 39.5, 40.3, 79.5, 79.6, 155.9, 155.8, 172.6 ppm; IR: v_{max} = 3353, 2970, 2936, 2868, 1682, 1640, 1517, 1446, 1388, 1364, 1298, 1270, 1249, 1162, 1102, 999, 984, 870, 837; HRMS (ESI): calcd. for $C_{22}H_{43}N_3O_5$ 429.3203; found 429.3200.

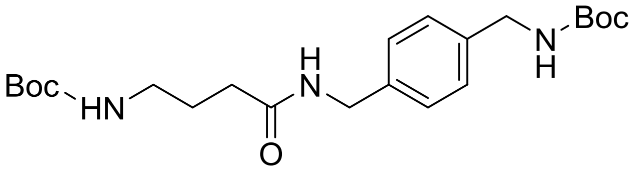

4-(*tert*-Butyloxycarbonylamino)-*N*-(4-(*tert*-butyloxycarbonylamino)benzyl)butanamide (**6f**): The compound was prepared according to GP 3 yielding 88% (2.96 g) of **6f** as a white solid, mp 124–126 °C. ^1H NMR (300 MHz, $CDCl_3$) δ 1.41–1.43 (s, 9H), 1.44–1.46 (s, 9H), 1.76–1.88 (q, J = 6.7 Hz, 2H), 2.20–2.28 (t, 4H, J = 7.1 Hz), 3.10–3.20 (m, 2H), 4.24–4.30 (d, J = 5.9 Hz, 2H), 4.38–4.44 (d, 4 H, J = 5.8 Hz), 4.79 (s, 1H), 4.85 (s, 1H), 6.45 (s, 1H), 7.23 (m, 4H) ppm; ^{13}C NMR (75 MHz, $CDCl_3$) δ 26.8, 27.4, 27.6, 27.7, 28.8, 34.4, 40.1, 43.9, 44.8, 80.0, 80.2, 128.4, 128.7, 138.79, 140.0, 158.6, 175.4, 175.5 ppm; IR v_{max} = 3340, 2973, 2936, 2882, 1678, 1643, 1531, 1463, 1445, 1389, 1364, 1266, 1208, 1100, 1052, 1027, 990, 897, 874 837, 804; HRMS (ESI): calcd. for $C_{22}H_{35}N_3O_5$ 421.2577; found 421.2576.

General procedure (GP 4) for the Boc-deprotection (**7a–f**): The Boc-protected diamines (**6a–f**) (1 mmol) were dissolved in EtOH (10 mL). Aqueous HCl (0.5%, 20 mL) was slowly added, and stirring was continued overnight. EtOH was evaporated, and the remaining H_2O was removed by lyophilisation. The residue was dissolved in warm EtOH (30 mL), the product was precipitated by careful addition of Et_2O and then centrifuged, and the supernatant was decanted off. Recrystallisation from MeOH and drying in vacuo afforded the deprotected amine hydrochlorides as colourless solids.

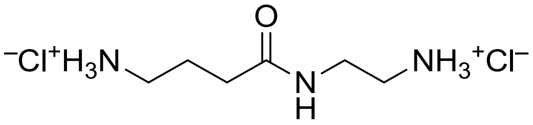

4-Amino-*N*-(2-aminoethyl)butanamide dihydrochloride (**7a**). The compound was prepared according to GP 4 yielding 60% (130 mg) of **7a** as a white solid, mp 144–146 °C. ^{1}H NMR (MeOD, 300 MHz) δ 1.85–1.99 (q, 2H), 2.38–2.47 (t, 2H, *J* = 7.2 Hz), 2.90 (t, 2H, *J* = 7.0 Hz), 3.08 (t, 2H, *J* = 7.3 Hz), 3.48 (t, 2H, *J* = 7.0 Hz) ppm; Amine and amide protons are not detected due to exchange effects with the solvent (5 H). ^{13}C NMR (DMSO, 75 MHz) δ 24.5, 24.7, 54.8, 113.2, 130.3, 174.4 ppm; IR: ν_{max} = 3430, 3251, 3085, 3053, 2944, 2383, 2335, 2032, 1779, 1682, 1611, 1539, 1482, 1386, 1349, 1213, 1152, 1068, 1019, 976, 909; HRMS (ESI): calcd. for $C_6H_{15}N_3O$ 145.1215; found 145.1217.

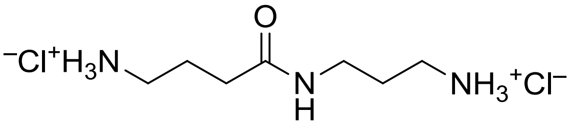

4-Amino-*N*-(3-aminopropyl)butanamide dihydrochloride (**7b**). The compound was prepared according to GP 4 yielding 45% (104 mg) of **7b** as a white solid, mp 140–142 °C. ^{1}H NMR (D_2O, 300 MHz) δ 1.72–1.91 (m, 4H), 2.28 (t, *J* = 7.2 Hz, 2H), 2.90 (m, 4H), 3.20 (t, *J* = 7.0 Hz, 2H) ppm. Amine and amide protons are not detected due to exchange effects with the solvent (5 H). ^{13}C NMR (DMSO, 75 MHz) δ 24.5, 33.8, 37.1, 54.2, 110.2, 124.4, 174.4 ppm; IR: ν_{max} = 3430, 3231, 3075, 3053, 2972, 2940, 2874, 2803, 2744, 2629, 1779,

1682, 1611, 1534, 1492, 1386, 1340, 1310, 1284, 1263, 1019, 976, 909; HRMS (ESI): calcd. for $C_7H_{17}N_3O$ 159.1371; found 159.1374.

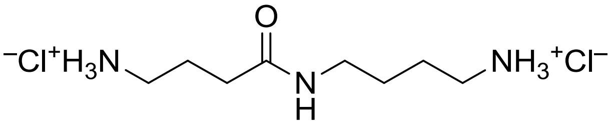

4-Amino-*N*-(4-aminobutyl)butanamide dihydrochloride (**7c**) [6]. The compound was prepared according to GP 4 yielding 55% (138 mg) of **7c**.

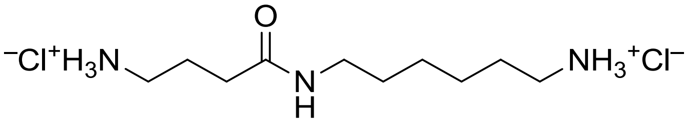

4-Amino-*N*-(6-aminohexyl)butanamide dihydrochloride (**7d**): The compound was prepared according to GP 4 yielding 50% (147 mg) of **7d** as a white solid, mp 147–150 °C. ^{1}H NMR (MeOD, 300 MHz) δ 1.34–1.48 (m, 4H), 1.49–1.60 (m, 2H), 1.62–1.74 (m, 2H), 1.87–1.99 (q, 2H, *J* = 7.3 Hz), 2.34–2.41 (t, 2H, *J* = 7.2 Hz), 2.87–3.01 (m, 4H), 3.16–3.23 (t, 2H, *J* = 7.0 Hz). Amine and amide protons are not detected due to exchange effects with the solvent (5 H). ^{13}C NMR (DMSO, 75 MHz) δ 24.5, 24.7, 30.5, 32.3, 33.8, 37.1, 48.2, 110.2, 130.3, 174.5 ppm; IR: ν_{max} = 3437, 3231, 3085, 3053, 2972, 2944, 2874, 2803, 2744, 2629, 2585, 2383, 2335, 2032, 1779, 1682, 1611, 1539, 1482, 1386, 1349, 1310, 1284, 1263, 1213, 1152, 1068, 1019, 976, 909; HRMS (ESI): calcd. for $C_{10}H_{23}N_3O$ 201.1841; found 201.1845.

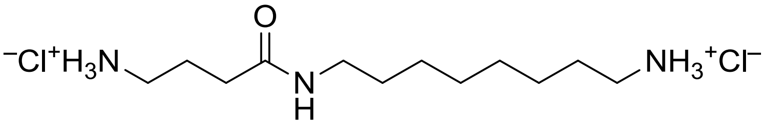

4-Amino-*N*-(8-aminooctyl)butanamide dihydrochloride (**7e**): The compound was prepared according to GP 4 yielding 40% (120 mg) of **7e** as a white solid, mp 136–138 °C. ^{1}H NMR (MeOD, 300 MHz) δ 1.30–1.45 (m, 8H), 1.46–1.58 (m, 2H), 1.59–1.72 (m, 2H), 1.86–1.98 (q, 2H, *J* = 7.3 Hz), 2.34–2.40 (t, 2H, *J* = 7.1 Hz), 2.87–3.00 (m, 4H), 3.14–3.22 (t, 2H, *J* = 7.1 Hz) ppm. Amine and amide protons are not detected due to exchange effects with the

solvent (5 H). ^{13}C NMR δ 24.6, 27.4, 27.9, 28.6, 30.0, 30.1, 33.8, 40.8, 49.1, 113.4, 127.8, 171.2 ppm; IR: v_{max} = 3347, 3329, 3126, 3080, 3046, 2973, 2941, 2892, 2862, 2813, 2740, 2380, 2175, 2020, 1681, 1611, 1540, 1484, 1384, 1349, 1311, 1284, 1262, 1240, 1197, 1152, 1065, 1011, 976, 939, 893, 839; HRMS (ESI): calcd. for $C_{12}H_{27}N_3O$ 229.2154; found 229.2160.

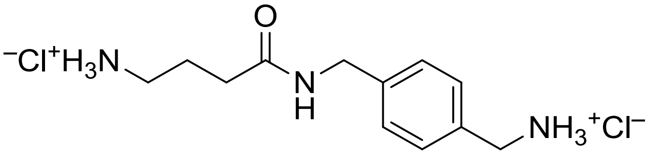

4-Amino-*N*-(4-(aminomethyl)benzyl)butanamide dihydrochloride (**7f**): The compound was prepared according to GP 4 yielding 50% (168 mg) of **7f** as a white solid, mp 251–253 °C. ^{1}H NMR (MeOD, 300 MHz) δ 1.89–2.01 (q, 2H, *J* = 7.3 Hz), 2.38–2.46 (t, 2H, *J* = 7.1 Hz), 2.94–3.01 (t, 2H, *J* = 7.4 Hz), 4.10 (s, 2H), 4.39 (s, 2H), 7.34–7.46 (m, 4H) ppm. Amine and amide protons are not detected due to exchange effects with the solvent (5 H). ^{13}C NMR (DMSO, 75 MHz) δ 24.5, 33.7, 40.5, 43.8, 44.2, 114.1, 125,3 129.3, 130.3, 133.4, 141.3, 174.5 ppm; IR: v_{max} = 3340, 3279, 2972, 2939, 2880, 2361, 2174, 2048, 1678, 1624, 1536, 1505, 1463, 1423, 1388, 1364, 1299, 1266, 1248, 1161, 1099, 1053, 1027, 989, 896, 875, 836; HRMS (ESI): calcd. for $C_{12}H_{19}N_3O$ 221.1528; found 221.1530.

I.II NMR and mass spectra

N,*N´*-(butane-1,4-diyl)bis(4-aminobutanamide) dihydrochloride (**4a**)

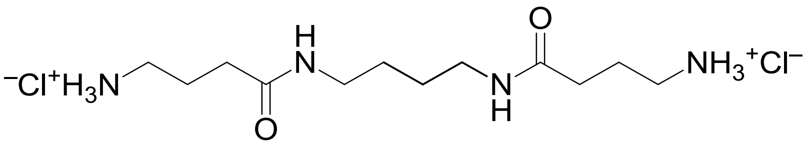

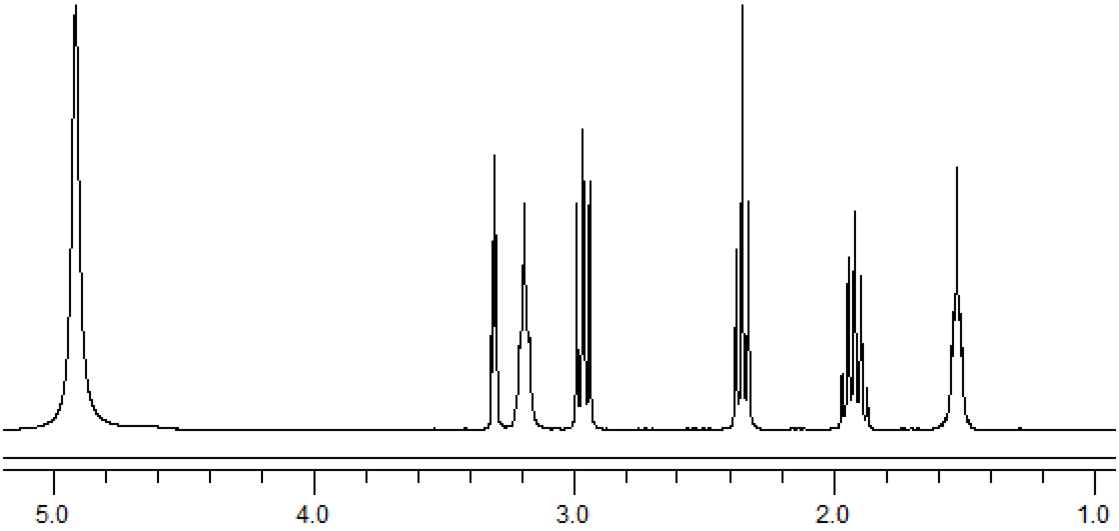

Figure S1: ^{1}H NMR (300 MHz, MeOD) of *N*,*N´*-(butane-1,4-diyl)bis(4-aminobutanamide) dihydrochloride (**4a**).

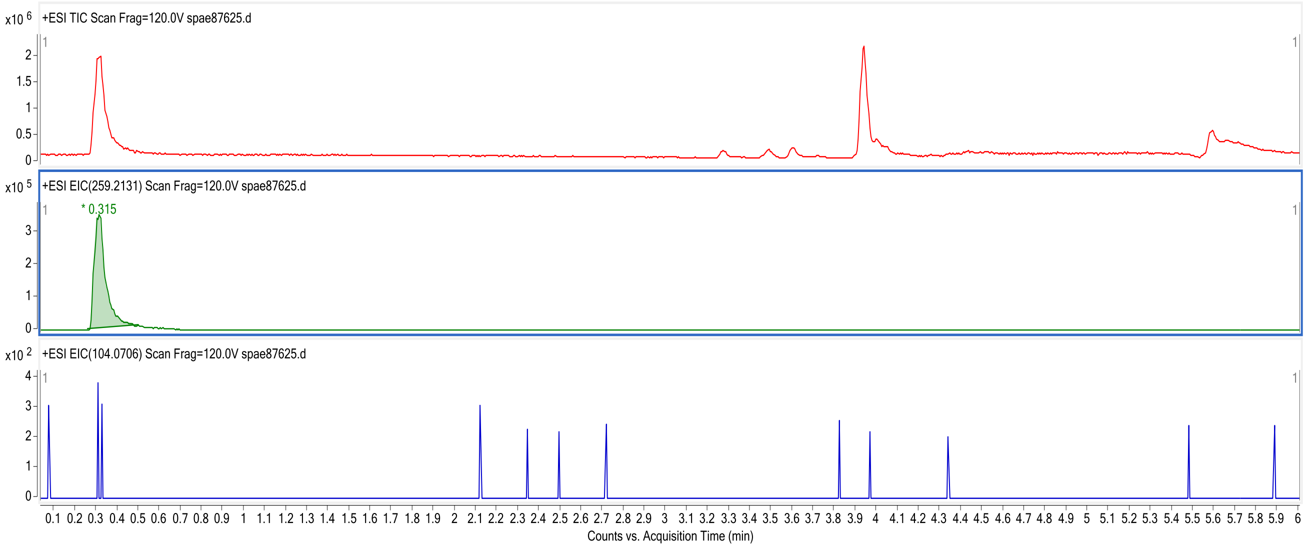

Figure S2: HPLC–MS of *N*,*N´*-(butane-1,4-diyl)bis(4-aminobutanamide) dihydrochloride (**4a**).

N,*N´*-(hexane-1,6-diyl)bis(4-aminobutanamide) dihydrochloride (**4b**)

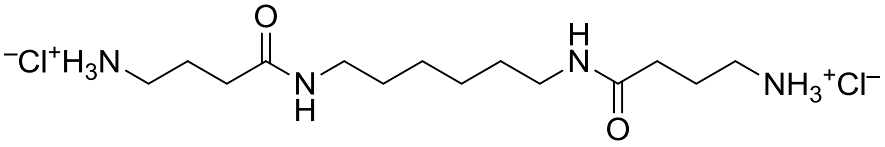

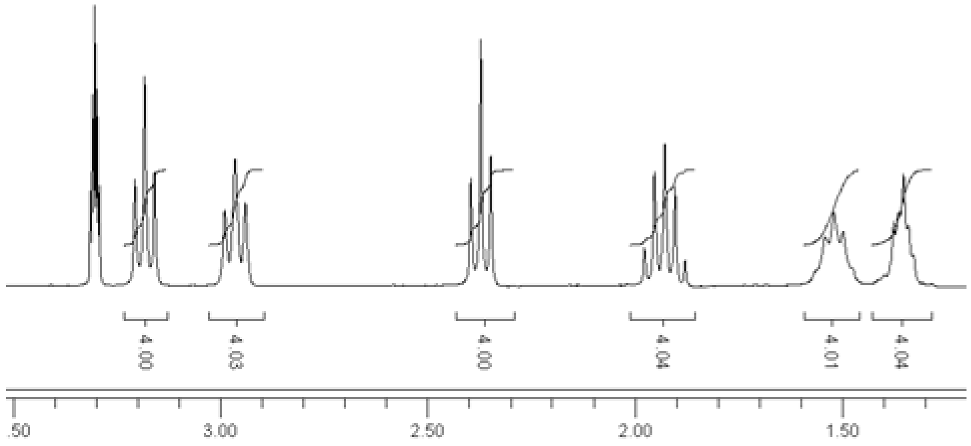

Figure S3: ^{1}H NMR (300 MHz, MeOD) of *N*,*N´*-(hexane-1,6-diyl)bis(4-aminobutanamide) dihydrochloride (**4b**).

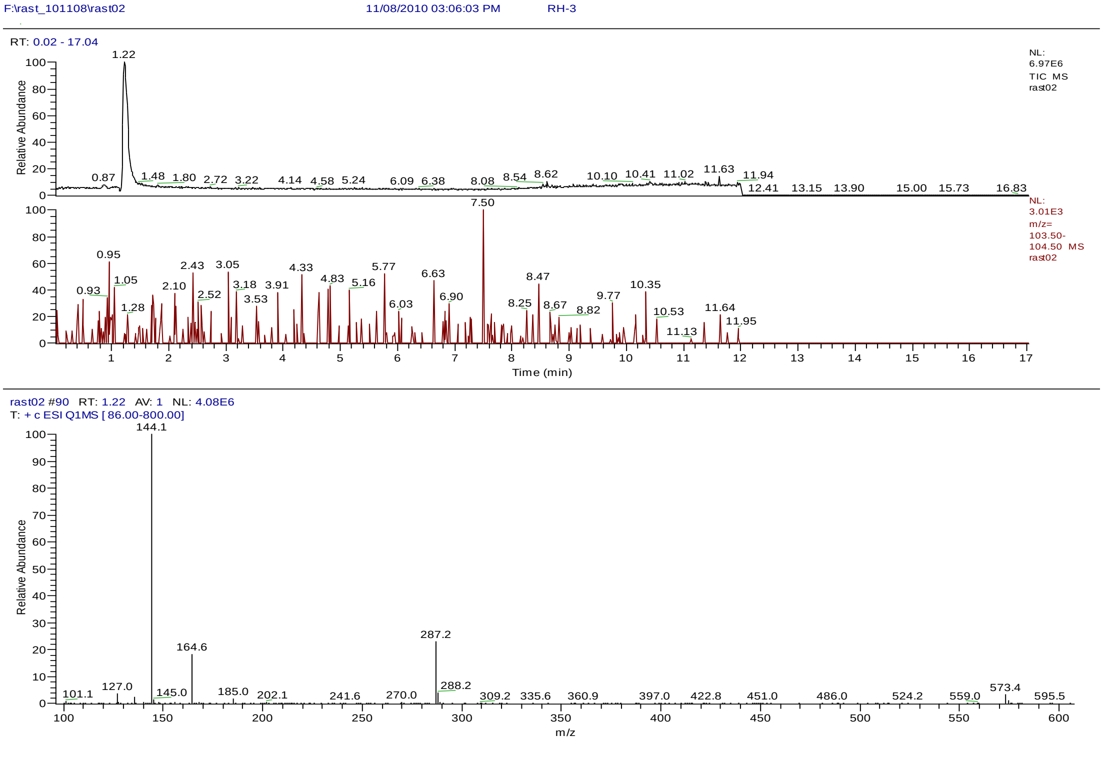

Figure S4: HPLC–MS of *N*,*N´*-(hexane-1,6-diyl)bis(4-aminobutanamide) dihydrochloride (**4b**).

N,*N*´-(octyl-1,8-diyl)bis(4-aminobutanamide) dihydrochloride (**4c**).

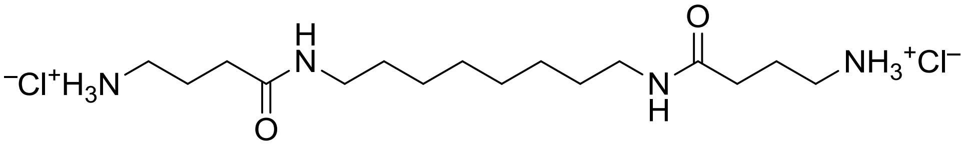

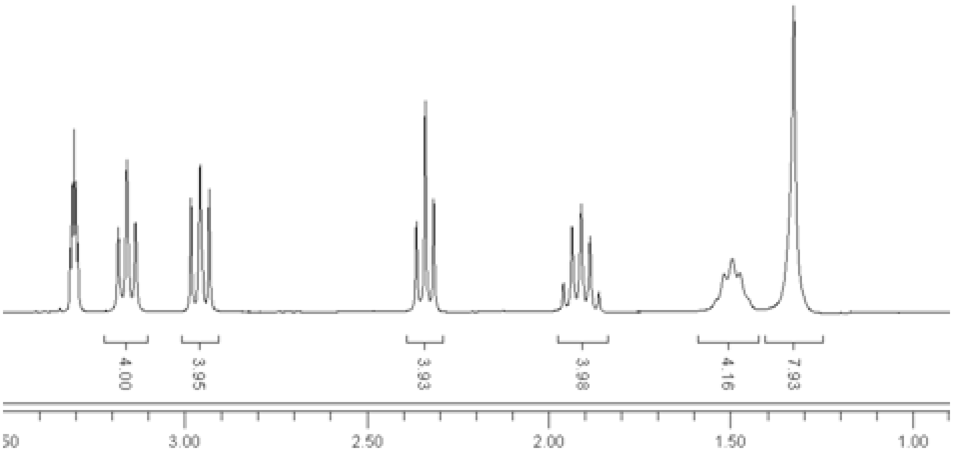

Figure S5: ^{1}H NMR (300 MHz, MeOD) of *N*,*N*´-(octyl-1,8-diyl)bis(4-aminobutanamide) dihydrochloride (**4c**).

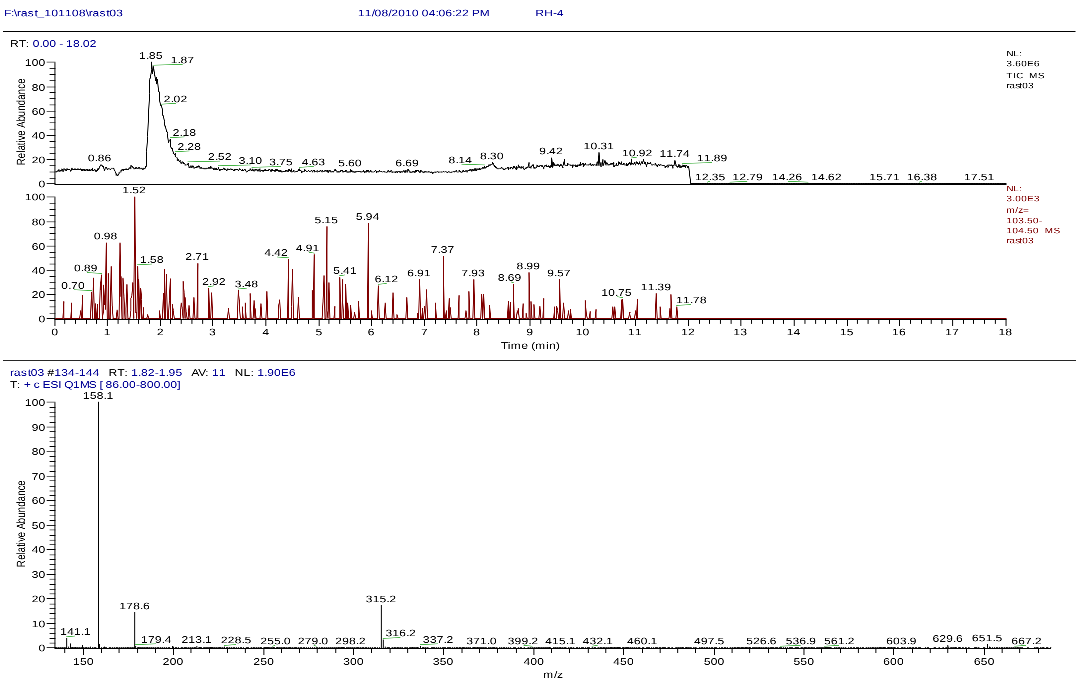

Figure S6: HPLC–MS of *N*,*N*´-(octyl-1,8-diyl)bis(4-aminobutanamide) dihydrochloride (**4c**).

4-Amino-*N*-(2-aminoethyl)butanamide dihydrochloride (**7a**).

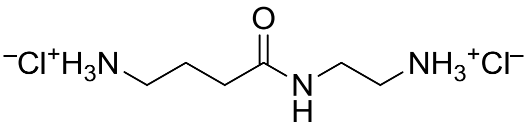

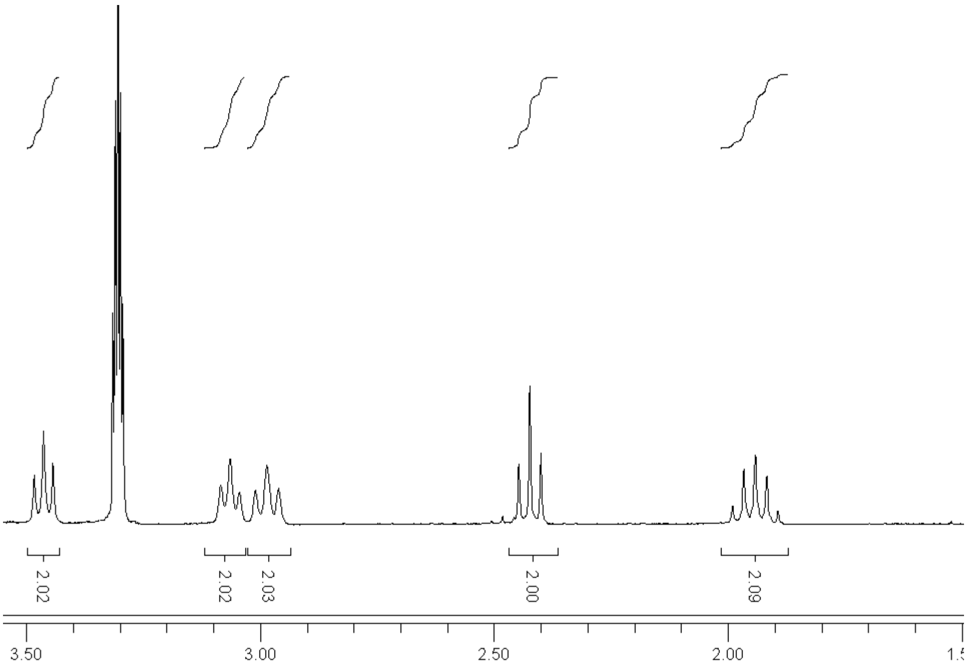

Figure S7: ^{1}H NMR (300 MHz, MeOD) of 4-amino-*N*-(2-aminoethyl)butanamide dihydrochloride (**7a**).

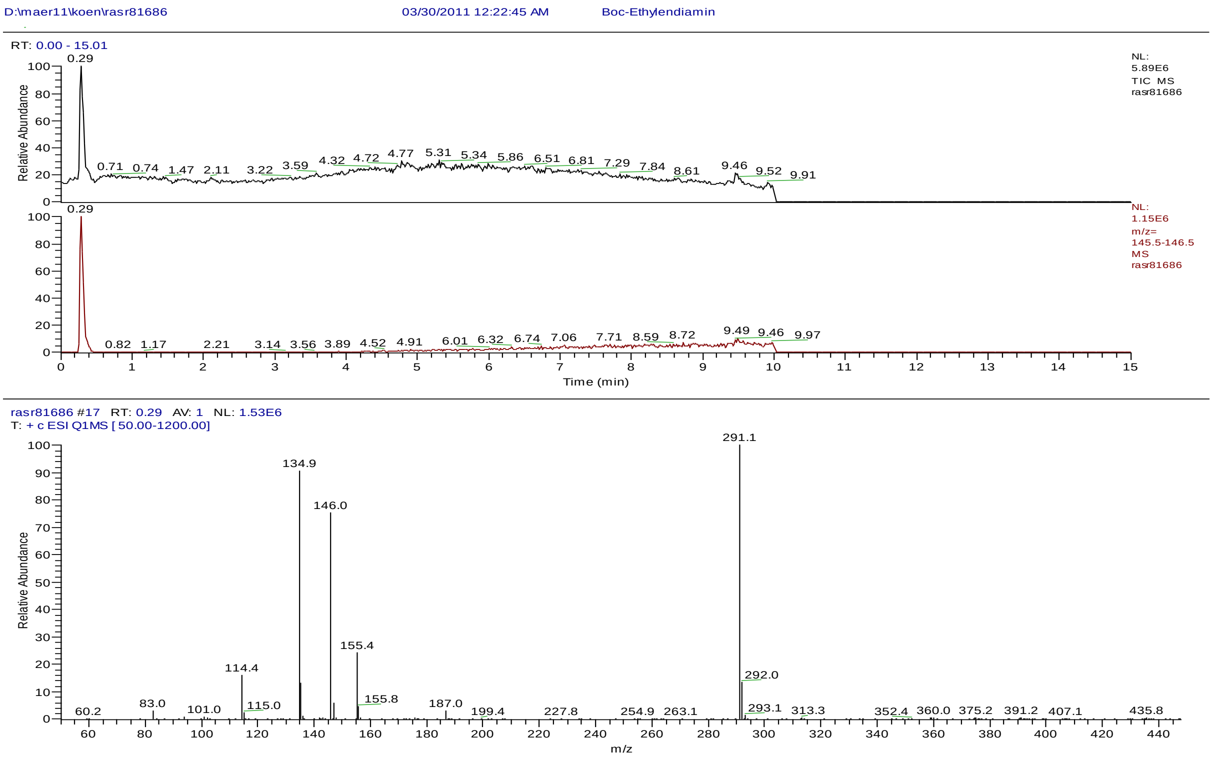

Figure S8: HPLC–MS of 4-amino-*N*-(2-aminoethyl)butanamide dihydrochloride (**7a**).

4-Amino-*N*-(2-aminopropyl)butanamide dihydrochloride (**7b**).

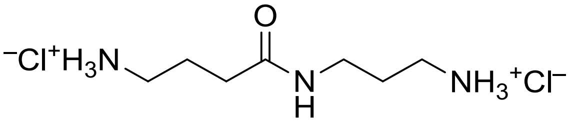

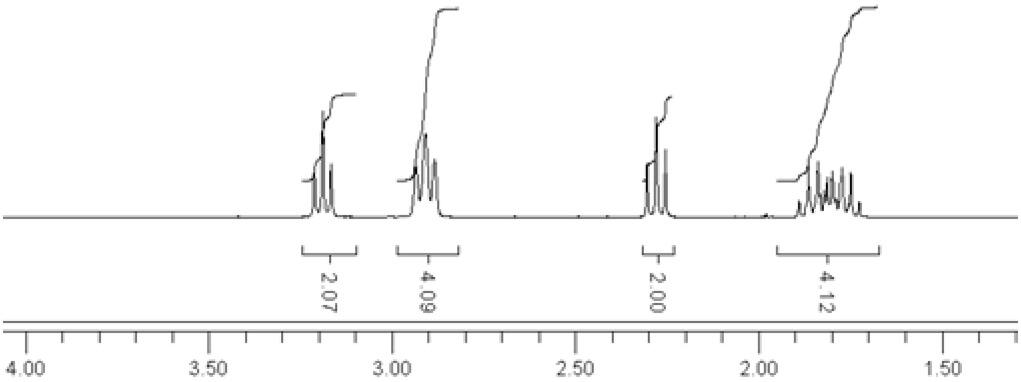

Figure S9: ^{1}H NMR (300 MHz, D_2O) of 4-amino-*N*-(3-aminopropyl)butanamide dihydrochloride (**7b**).

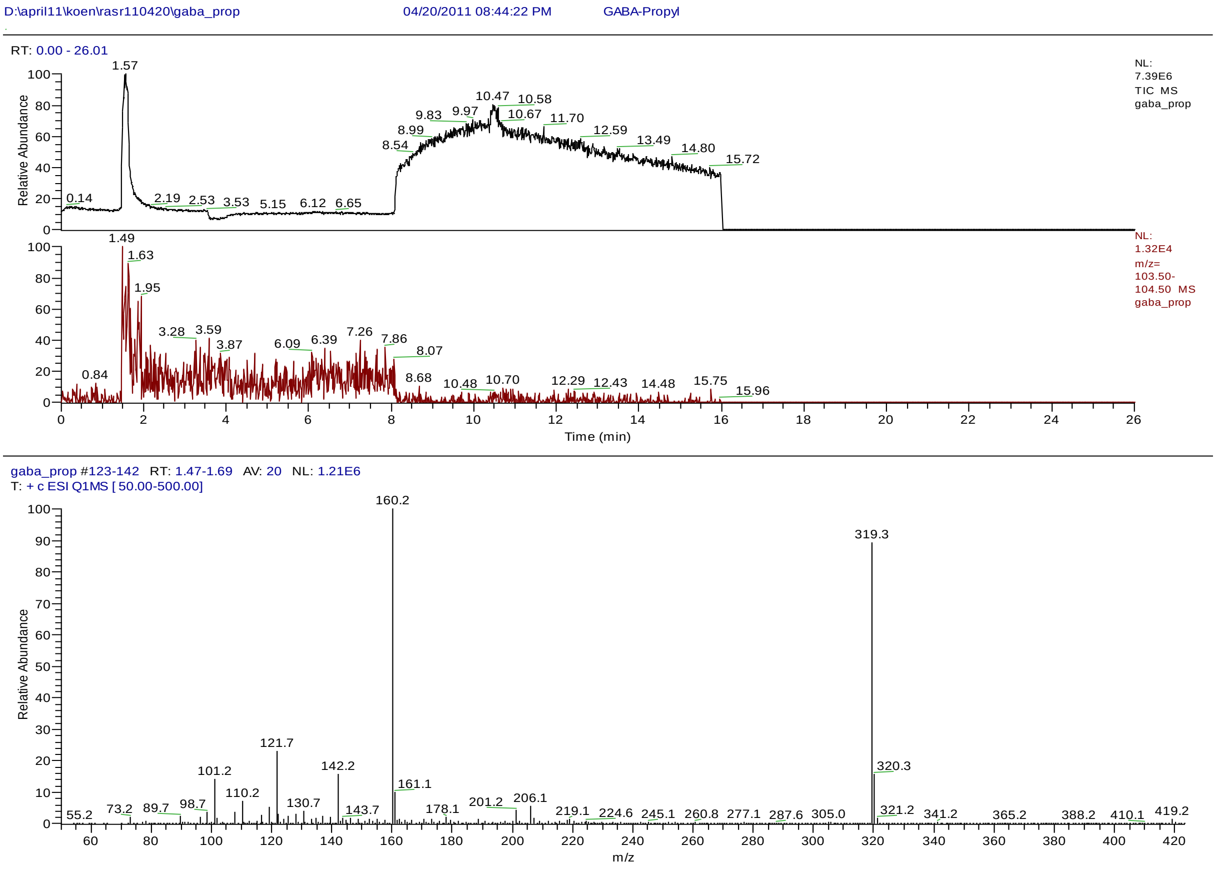

Figure S10: HPLC–MS of 4-amino-*N*-(3-aminopropyl)butanamide dihydrochloride (**7b**).

4-Amino-*N*-(4-aminobutyl)butanamide dihydrochloride (**7c**).

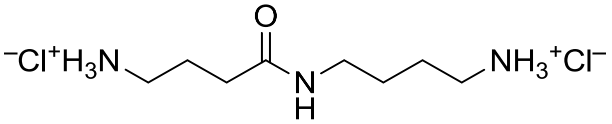

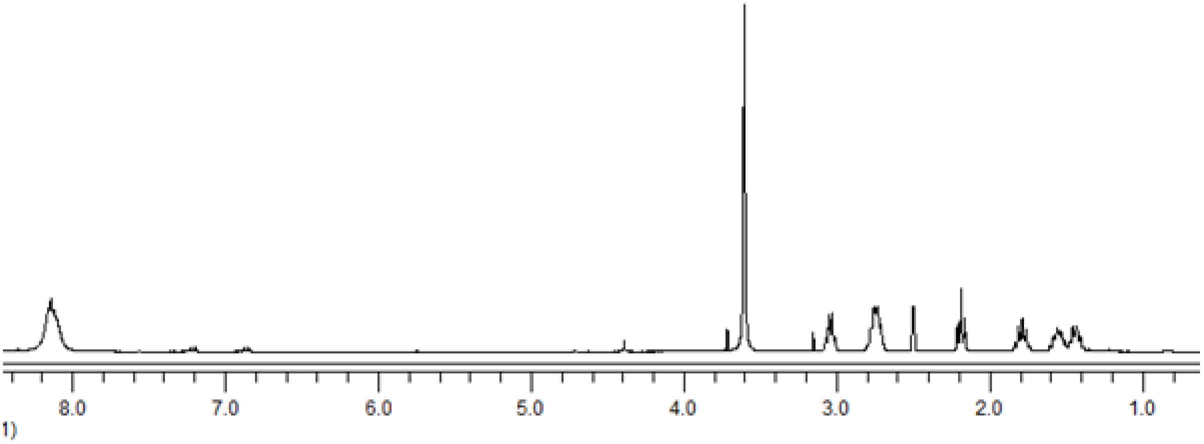

Figure S11: ^{1}H NMR (300 MHz, DMSO) of 4-amino-*N*-(4-aminobutyl)butanamide dihydrochloride (**7c**).

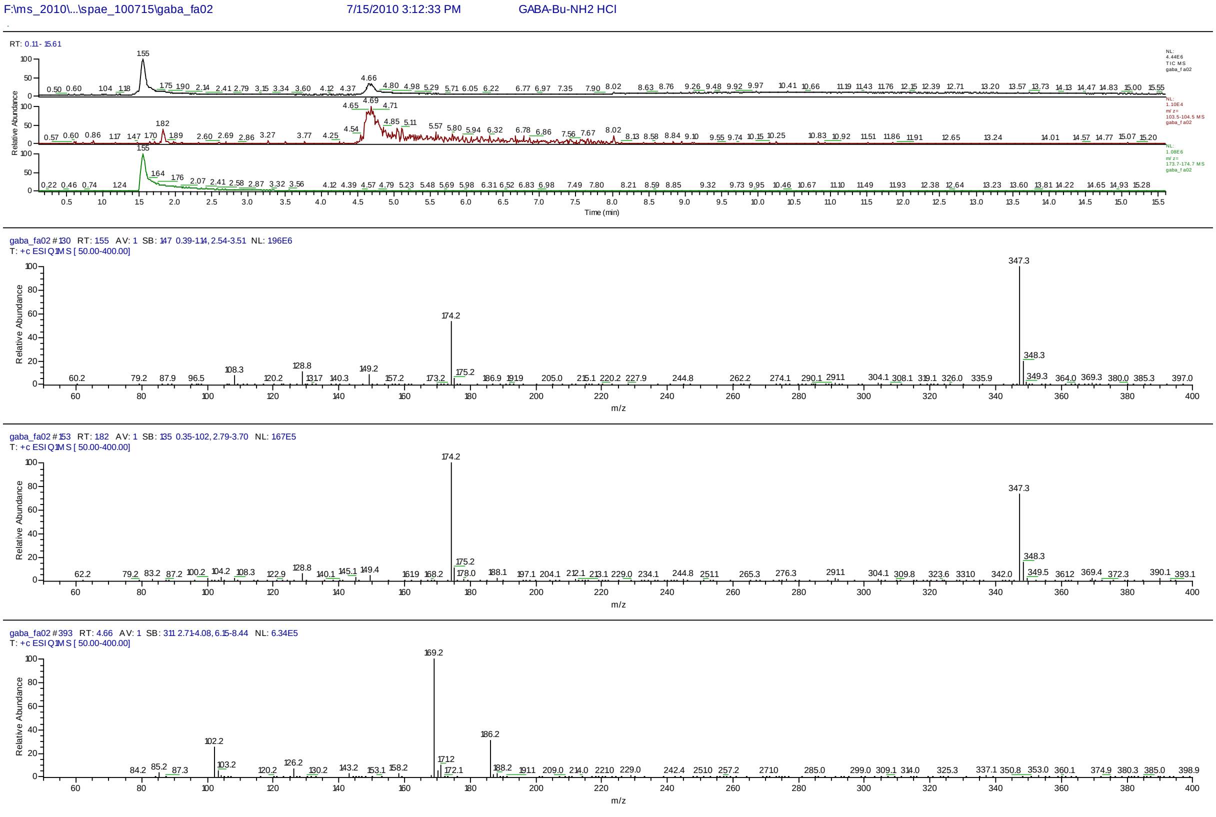

Figure S12: HPLC–MS of 4-amino-*N*-(4-aminobutyl)butanamide dihydrochloride (**7c**).

4-Amino-*N*-(6-aminohexyl)butanamide-hydrochloride (**7d**).

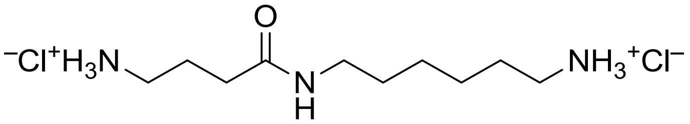

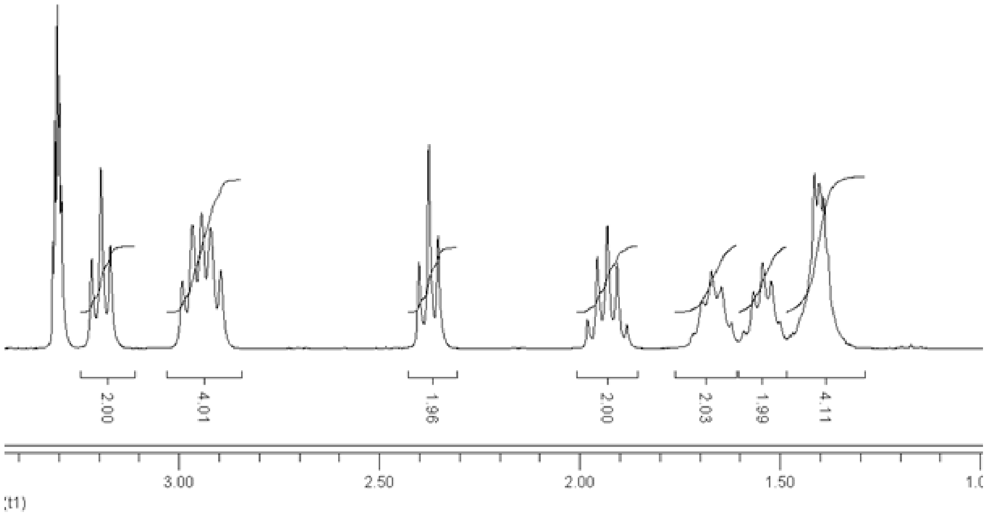

Figure S13: ^{1}H NMR (300 MHz, MeOD) of 4-amino-*N*-(6-aminohexyl)butanamide dihydrochloride (**7d**).

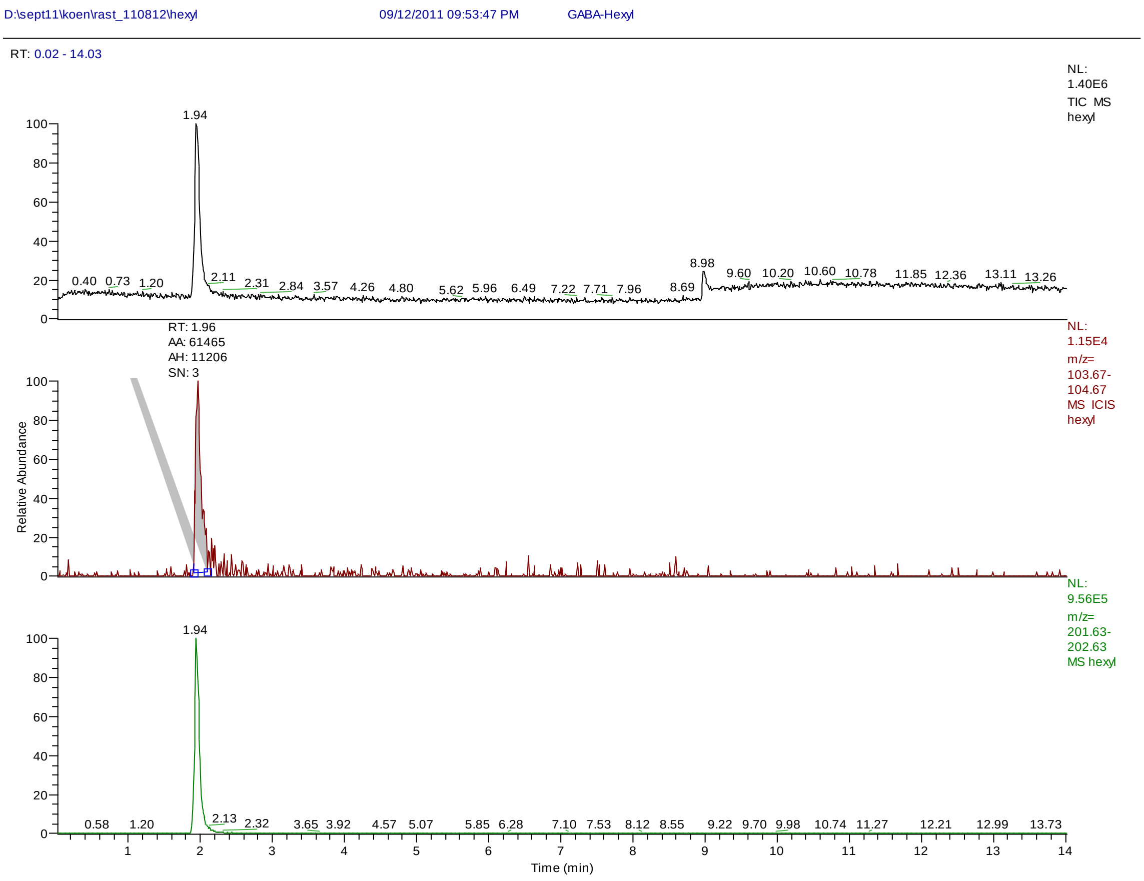

Figure S14: HPLC–MS of 4-amino-*N*-(6-aminohexyl)butanamide dihydrochloride (**7d**).

4-Amino-*N*-(8-aminooctyl)butanamide dihydrochloride (**7e**).

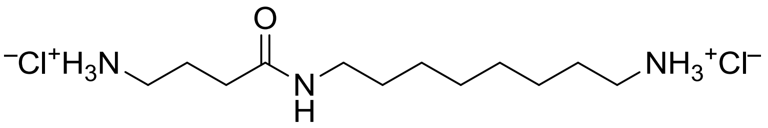

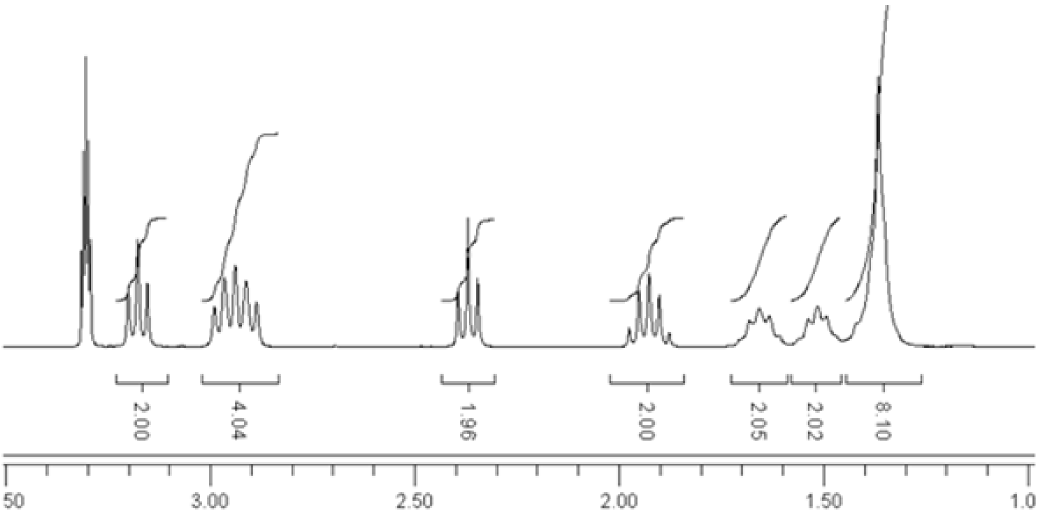

Figure S15: ^{1}H NMR (300 MHz, MeOD) of 4-amino-*N*-(8-aminooctyl)butanamide dihydrochloride (**7e**).

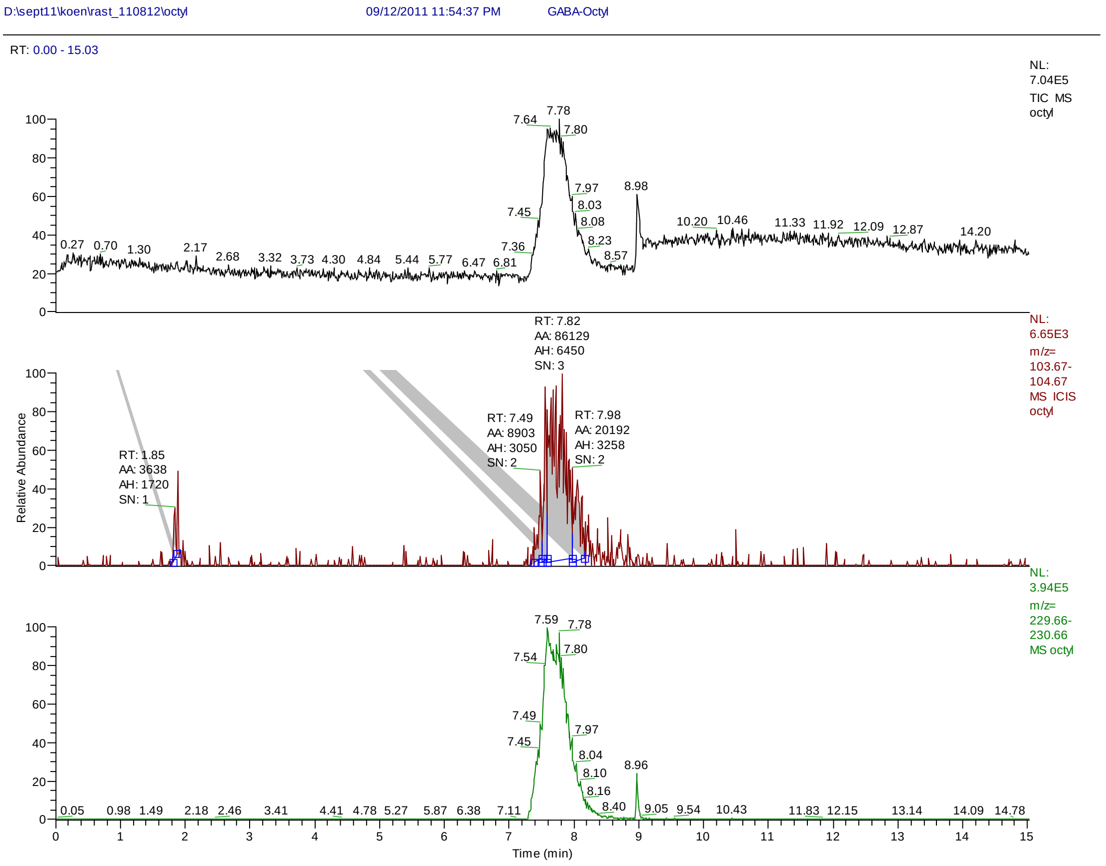

Figure S16: HPLC–MS of 4-amino-*N*-(8-aminooctyl)butanamide dihydrochloride (**7e**).

4-Amino-*N*-(4-(aminomethyl)benzyl)butanamide dihydrochloride (**7f**).

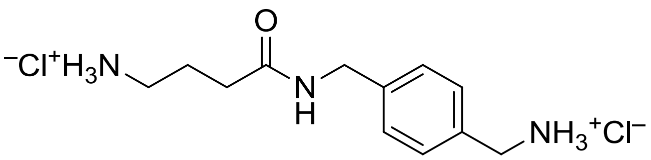

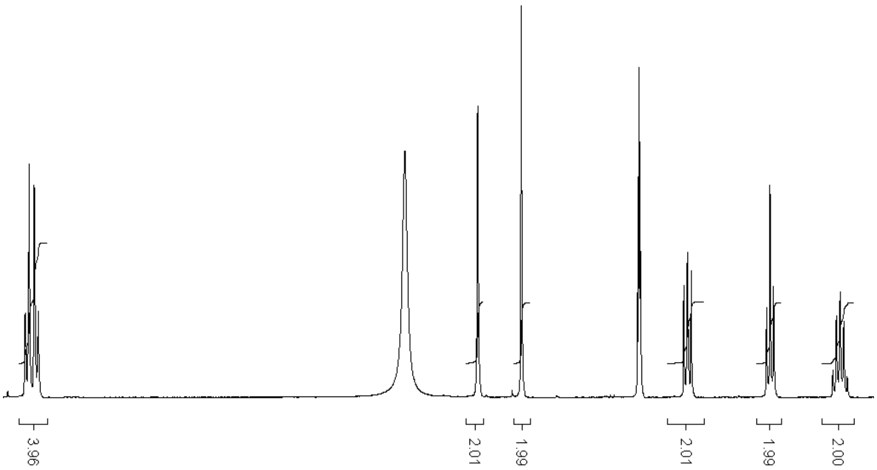

Figure S17: ^{1}H NMR (300 MHz, MeOD) of 4-amino-*N*-(4-(aminomethyl)benzyl)butanamide dihydrochloride (**7f**).

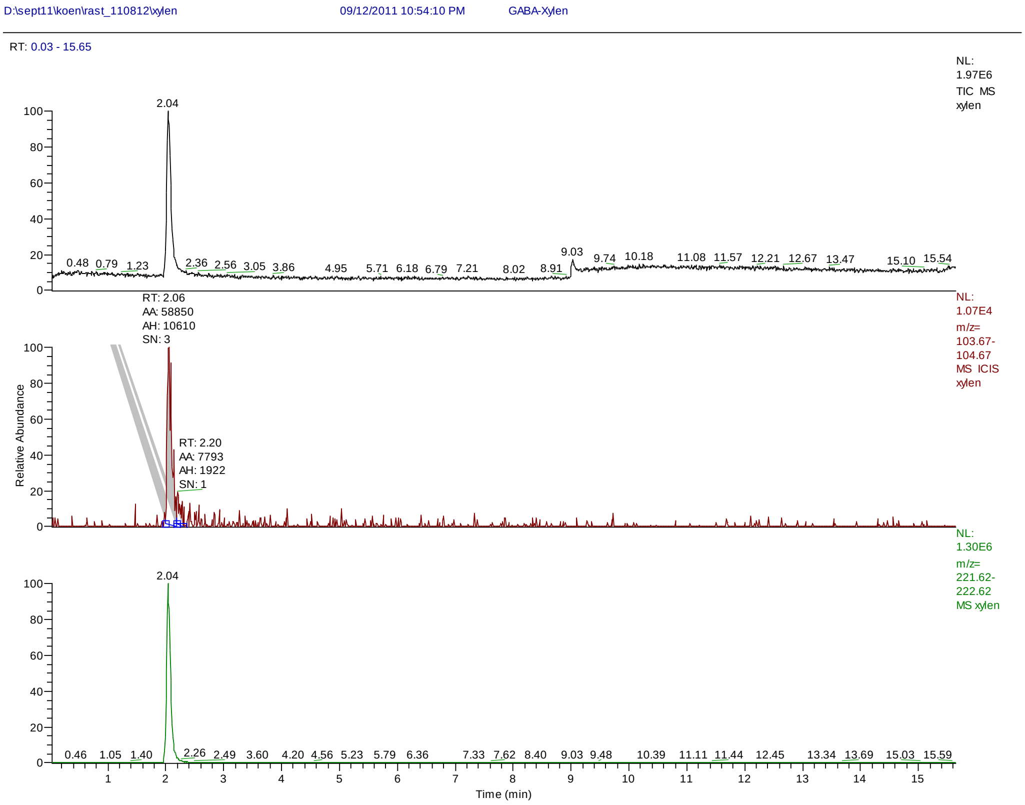

Figure S18: HPLC–MS of 4-amino-*N*-(aminomethyl)benzyl)butanamide dihydrochloride (**7f**).

Detection limit of GABA

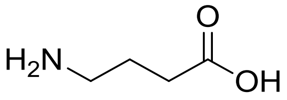

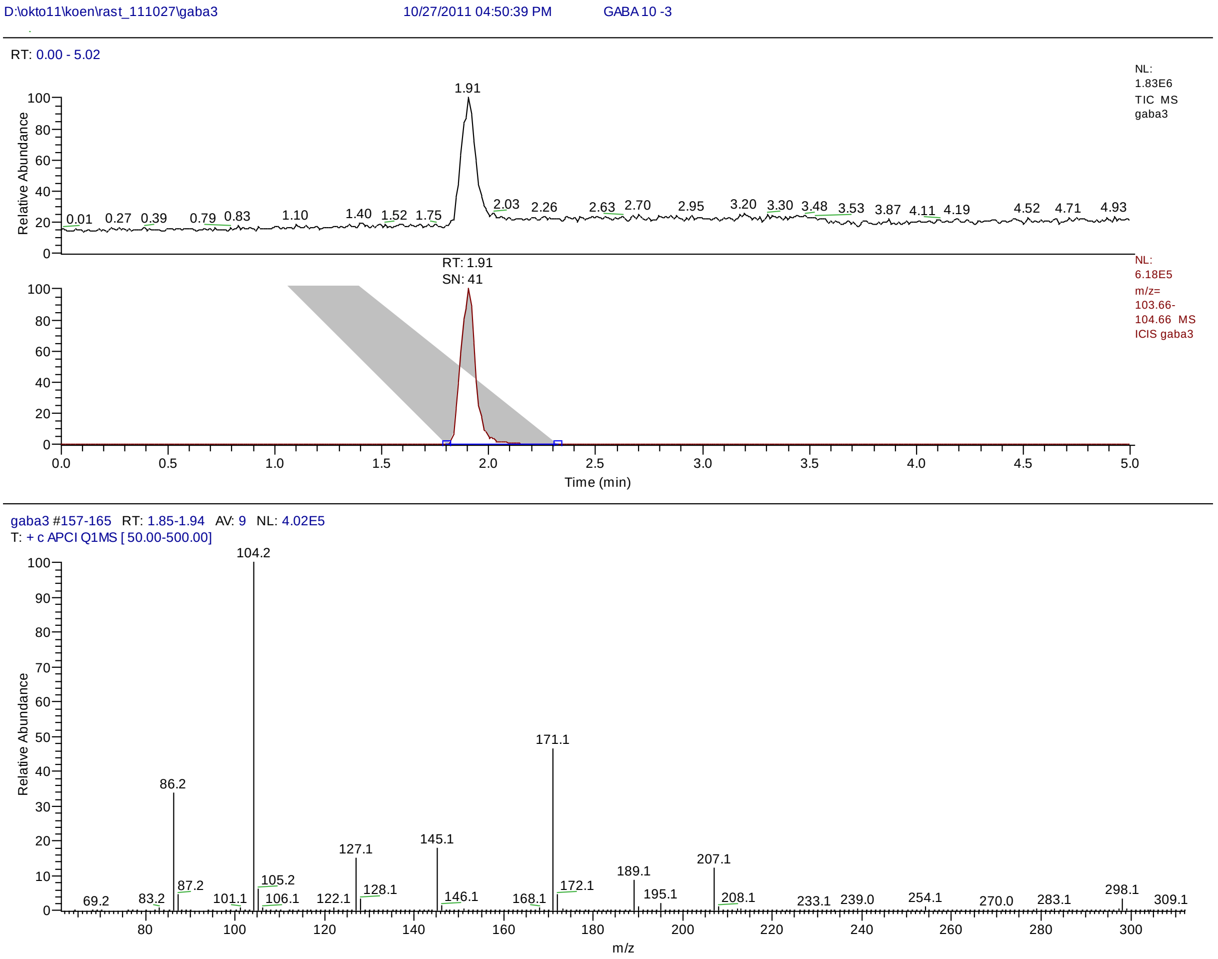

Figure S19: HPLC–MS of GABA (10^{-9} mol/L).

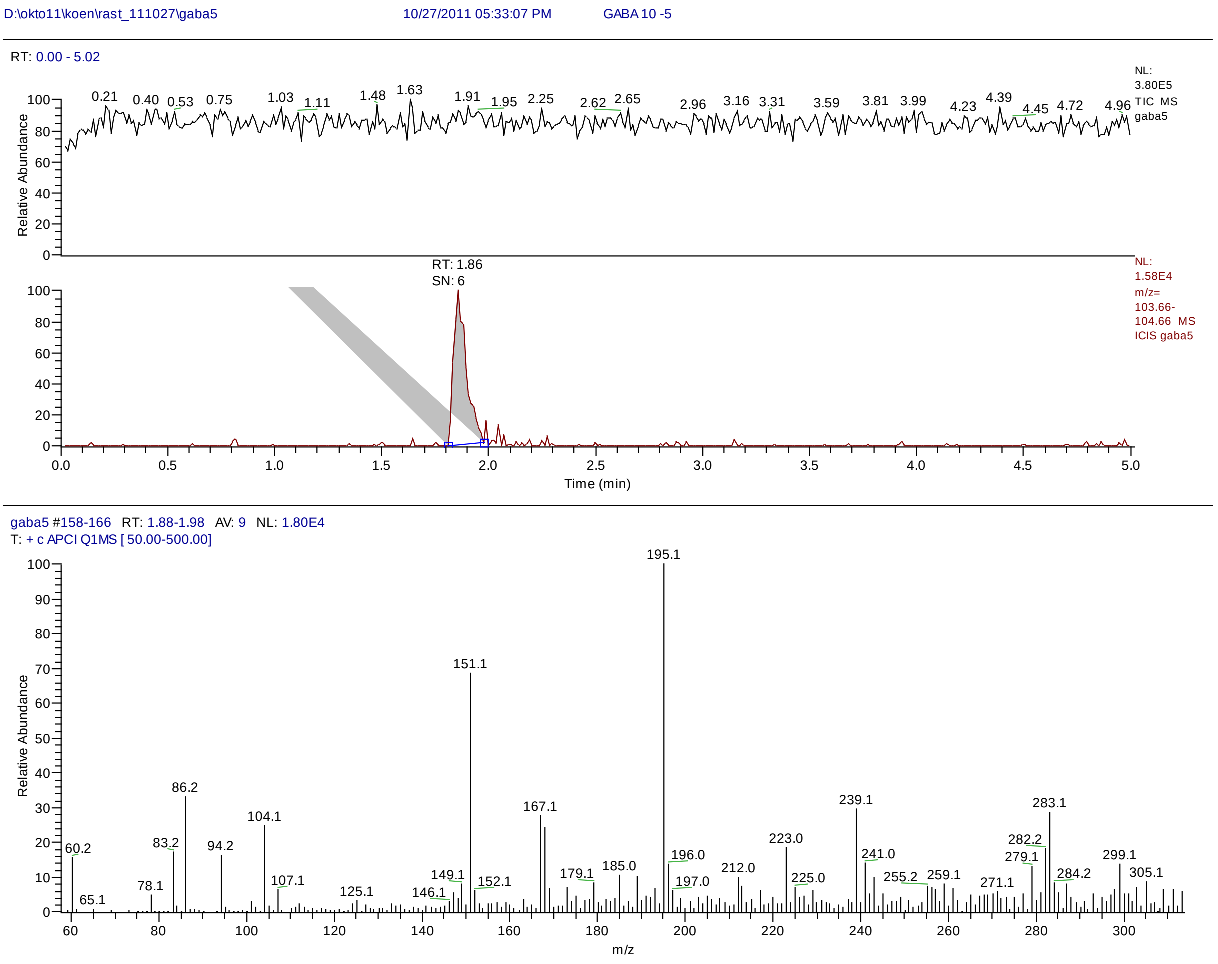

Figure S20: HPLC–MS GABA (10^{-11} mol/L).

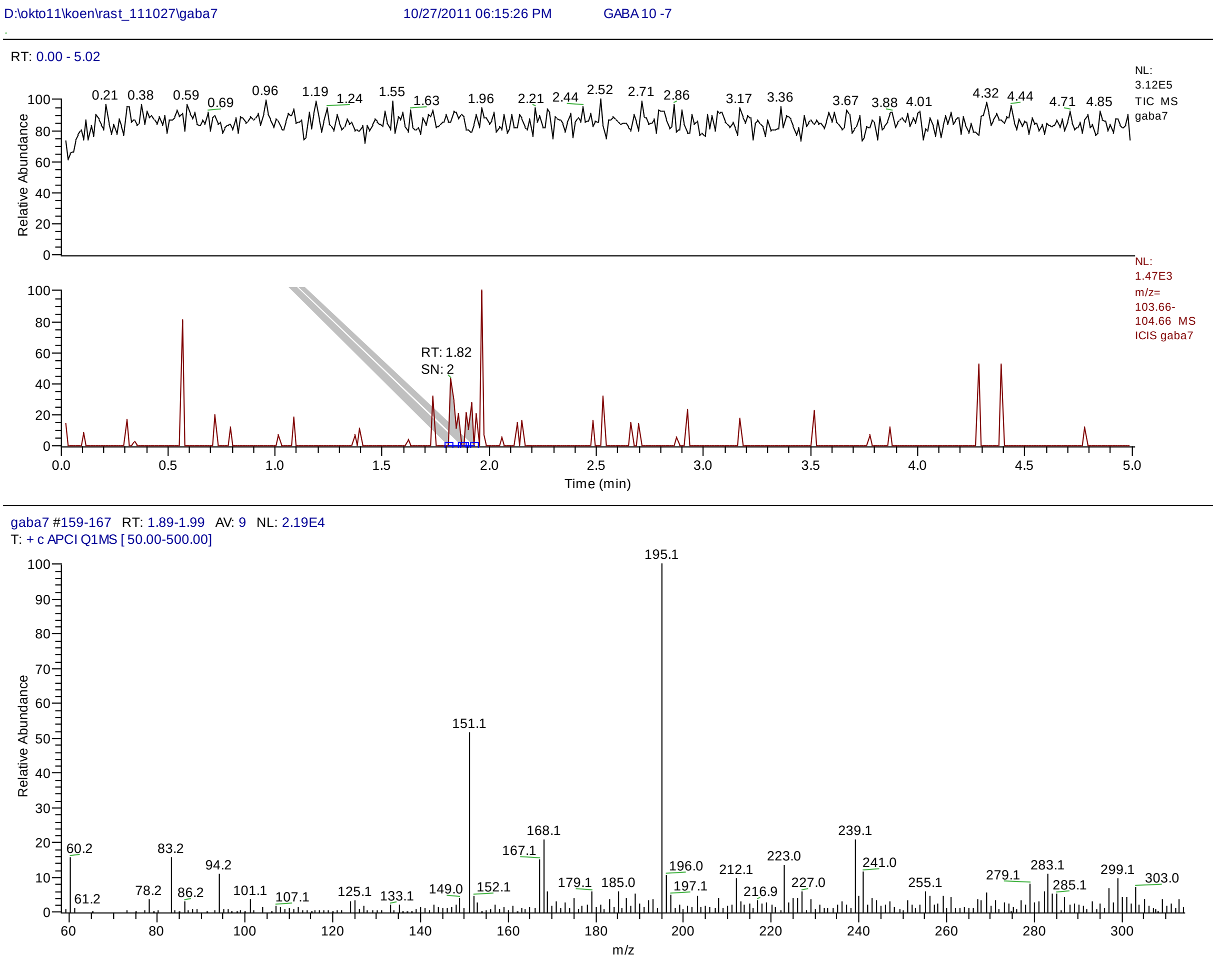

Figure S21: HPLC–MS GABA (10^{-13} mol/L).

II. Biology

II.I. Methods

Cell culture and transient transfection

Chinese Hamster Ovary (CHO-K1) cells were obtained from the American Type Culture Collection (ATCC, Molsheim, France) and maintained as previously described [7,8]. One day before the transfection, cells were plated on the coverslips (12–14 mm in diameter), which were placed inside 35 mm cell culture dishes with 2 mL of medium. CHO cells were transfected with a total 3 µg cDNA of α1-GFP + β2 + $\gamma 2_{Long}$ (ratio 1:1:1) using the Lipofectamine 2000 transfection protocol (Life Technology, USA). Three hours after the initial exposure of the cells to the cDNAs, a fresh cDNA-containing solution replaced the old one. Electrophysiological recordings were performed from fluorescent cells 48–72 hours after transfection.

Electrophysiological recordings

Whole-cell recordings were conducted on CHO-K1 cells at room temperature (20–25 °C) using an EPC-9 amplifier (HEKA Elektronik, Germany). Cells were continuously superfused with external solution containing (mM): NaCl 140, $CaCl_2$ 2, KCl 2.8, $MgCl_2$ 1, HEPES 20, glucose 10; pH 7.3; 320–330 mOsm. The patch pipette solution contained (mM): CsCl 140, $MgCl_2$ 2, MgATP 2, NaGTP 0.4, HEPES/CsOH 10, BAPTA/KOH 20; pH 7.3; 290 mOsm. Pipettes were pulled from borosilicate glass capillaries (Harvard Apparatus Ltd, USA) and had resistances of 5–8 MΩ.

For rapid replacement of solutions, a system of two parallel rectangular tubes, 100 µm in diameter, located at a distance of 40–50 µm from the tested cell, was used. The movement of tubes was controlled by a computer-driven fast exchange system (SF 77A Perfusion Fast-Step, Warner, USA). As measured by open tip electrode controls (1/10 NaCl), in this system a 20–80% solution exchange time was within 2–3 ms. Cells with a low input resistance (<150 MΩ) were excluded from analysis.

For quantitative estimations of agonist action, dose-response relationships were fitted by the equation

$$I = I_{max} / (1 + (EC_{50}/ [C])^{n})$$

where I is the current amplitude induced by the agonist at concentration [C], I_{max} is the maximum response of the cell, n is the Hill coefficient and EC_{50} is the concentration for which a half-maximum response is induced. Concentration-response relationships were constructed using at least four points.

III. References

1. Harrison, T. J.; Dake, G. R. *J. Org. Chem.* 2005, *70,* 10872–10874. doi:10.1021/jo051940a
2. Eisenführ, A.; Arorab, P. S.; Sengle, G.; Takaoka, L. R.; Nowick, J. S.; Famulok, M. *Bioorg. Med. Chem.* 2003, *11,* 235–249. doi:10.1016/S0968-0896(02)00311-5
3. Cinelli, M. A.; Cordero, B.; Dexheimer, T. S.; Pommier, Y.; Cushman, M. *Bioorg. Med. Chem.* 2009, *17,* 7145–7155. doi:10.1016/j.bmc.2009.08.066
4. West, C. W.; Estiarte, M. A.; Rich, D. H. *Org. Lett.* 2001, *3,* 1205–1208. doi:10.1021/ol015678d
5. Chen, G.; Lean, J. T.; Alcalá, M.; Mallouk, T. E. *J. Org. Chem.* 2001, *66,* 3027–3034. doi:10.1021/jo991054y
6. Carlier, P. R.; Chow, E. S.-H.; Barlow, R. L.; Bloomquist, J. R. *Bioorg. Med. Chem. Lett.* 2002, *12,* 1985–1988. doi:10.1016/S0960-894X(02)00299-8
7. Medina, I.; Krapivinsky, G.; Arnold, S.; Kovoor, P.; Krapivinsky, L.; Clapham, D. E. *J. Biol. Chem.* 2000, *275,* 29709–29716. doi:10.1074/jbc.M004989200
8. Waseem, T.; Mukhtarov, M.; Buldakova, S.; Medina, I.; Bregestovski, P. *J. Neurosci. Methods* 2010, *193,* 14–23. doi:10.1016/j.jneumeth.2010.08.002
